# Cytosol Peroxiredoxin and Cell Surface Catalase Differentially Respond to H_2_O_2_ Stress in *Aspergillus nidulans*

**DOI:** 10.3390/antiox12071333

**Published:** 2023-06-23

**Authors:** Yunfeng Yan, Xiaofei Huang, Yao Zhou, Jingyi Li, Feiyun Liu, Xueying Li, Xiaotao Hu, Jing Wang, Lingyan Guo, Renning Liu, Naoki Takaya, Shengmin Zhou

**Affiliations:** 1 State Key Laboratory of Bioreactor Engineering, School of Biotechnology, East China University of Science and Technology, Shanghai 200237, China; 2 Faculty of Life and Environmental Sciences, University of Tsukuba, Tsukuba 305-8572, Japan

**Keywords:** peroxiredoxin, catalase, oxidative stress, hydrogen peroxide, *Aspergillus nidulans*

## Abstract

Both catalase and peroxiredoxin show high activities of H_2_O_2_ decomposition and coexist in the same organism; however, their division of labor in defense against H_2_O_2_ is unclear. We focused on the major peroxiredoxin (PrxA) and catalase (CatB) in *Aspergillus nidulans* at different growth stages to discriminate their antioxidant roles. The dormant conidia lacking PrxA showed sensitivity to high concentrations of H_2_O_2_ (>100 mM), revealing that PrxA is one of the important antioxidants in dormant conidia. Once the conidia began to swell and germinate, or further develop to young hyphae (9 h to old age), PrxA-deficient cells (Δ*prxA*) did not survive on plates containing H_2_O_2_ concentrations higher than 1 mM, indicating that PrxA is an indispensable antioxidant in the early growth stage. During these early growth stages, absence of CatB did not affect fungal resistance to either high (>1 mM) or low (<1 mM) concentrations of H_2_O_2_. In the mature hyphae stage (24 h to old age), however, CatB fulfills the major antioxidant function, especially against high doses of H_2_O_2_. PrxA is constitutively expressed throughout the lifespan, whereas CatB levels are low in the early growth stage of the cells developing from swelling conidia to early growth hyphae, providing a molecular basis for their different contributions to H_2_O_2_ resistance in different growth stages. Further enzyme activity and cellular localization analysis indicated that CatB needs to be secreted to be functionalized, and this process is confined to the growth stage of mature hyphae. Our results revealed differences in effectiveness and timelines of two primary anti-H_2_O_2_ enzymes in fungus.

## 1. Introduction

Catalases and peroxiredoxins (Prxs) are the two key antioxidants involved in H_2_O_2_ detoxification. Catalases decompose two molecules of H_2_O_2_ into H_2_O and oxygen [1]. Most Prxs reduce H_2_O_2_ to H_2_O with the concomitant oxidation of two cysteine residues to form a disulfide bond [2,3]. The contribution of Prxs and catalases to H_2_O_2_ resistance varies across organisms. In animals, almost all of H_2_O_2_ is enzymatically detoxified by glutathione peroxidase (Gpx) and catalase is of little import [4]. Similarly, loss of Gpx, but not catalase, induced sensitivity to H_2_O_2_ in *Saccharomyces cerevisiae* [5,6,7]. In *Escherichia coli*, the Prx AhpC efficiently scavenges low concentrations of H_2_O_2_, whereas catalase predominantly protects the cells at high H_2_O_2_ concentrations [8,9]. In *Schizosaccharomyces pombe*, the Prx Tpx1 is the first line of defense, controlling H_2_O_2_ generation during aerobic metabolism, whereas catalase does the same at high levels of H_2_O_2_ [10]. Contrary to all these organisms, the catalase KatG is the primary detoxifier of H_2_O_2_ produced during aerobic metabolism in *Bradyrhizobium japonicum* [11]. Clearly, the primary H_2_O_2_ detoxifying enzyme varies in a species-specific manner.

The antioxidant systems in filamentous fungi have been intensively studied, especially in *Aspergillus* species. PrxA has been identified as an indispensable antioxidant among the multiple Prxs in *Aspergillus nidulans* [12,13,14]. H_2_O_2_ degradation by PrxA requires thioredoxin and its reductase, along with NADPH consumption [12]. *A. nidulans* also possesses multiple catalases, including CatA, CatB, CatC, and CatD [15]. CatA is a spore-specific catalase whose mutation renders conidia sensitive to H_2_O_2_ [16]. CatB is present during the cell growth stages and protects against exogenous H_2_O_2_ [17]. CatC and CatD functions have not been elucidated, but they do not seem to be involved in H_2_O_2_ detoxification [15]. The individual PrxA and catalase families’ contributions to H_2_O_2_ detoxification in this fungus, and the extent of functional overlap, are unknown.

This study aimed to discriminate the functions of the fungal PrxA and the major catalase CatB of *A. nidulans* in protecting against H_2_O_2_. We compared the phenotypes of mutants after H_2_O_2_ exposure during the fungal lifespan from dormant conidia to mature hyphae and found the temporal and spatial differences in the functions of PrxA and CatB in *A. nidulans*.

## 2. Materials and Methods

### 2.1. Strains and Growth Conditions

Supplementary Materials Table S1 lists the *A. nidulans* strains used in this study. All fungal strains were grown at 37 °C in MM (1% glucose, 10 mM NaNO_3_, 7 mM KCl, 10 mM KH_2_PO_4_, 2 mM MgSO_4_, 2 mL/L Hunter’s trace metals, and pH 6.5) [18], and appropriately supplemented with 0.4 mg/L pyridoxine, 0.5 g/L uracil, 0.6 g/L uridine, and 0.4 mg/L biotin to meet the growth requirement. *E. coli* DH5α was used for molecular cloning.

### 2.2. Construction of Plasmids for Subsequent Recombinant Strain Construction

Table S2 lists the plasmids used in this study. Genomic DNA was extracted from *A. nidulans* A6 using the Genomic DNA Purification Kit (Promega, Madison, WI, USA). All plasmids were constructed using the ClonExpress MultiS One Step Cloning Kit (Vazyme, Nanjing, China), and PCR was performed using the PrimeSTAR HS DNA Polymerase (Takara, Osaka, Japan). The primers designed for constructing recombinant plasmids are listed in Table S3.

pUC19-*pyrG* is a modified pUC19 plasmid containing the selective marker gene *pyrG*, cloned from the genomic DNA of *A. nidulans* A6 into the multiple cloning site of XbaI. pUC19-*GFP*-*trpC*.T-*pyrG* is a pUC19-*pyrG*-based plasmid harboring the following sequences at the multiple cloning site: 5GA-linker-tagged green fluorescent protein (GFP) encoding sequence, *trpC* terminator, and *pyrG*. The pUC19-*pyrG* was linearized via an inverse PCR with the primer pair Inverse-1F/Inverse-1R. The DNA fragment for 5GA-linker-tagged GFP expression was cloned from our previously constructed plasmid pUC-*GFP* [14] with the primer pair *GFP*-F/*GFP*-R. The *trpC* terminator was cloned from the genomic DNA of *A. nidulans* A6 with the primer pair *trpC*.T-1F/*trpC*.T-1R. The three resulting DNA fragments were cyclized to construct pUC19-*GFP*-*trpC*.T-*pyrG* using the One Step Cloning Kit. pUC19-*GFP*-*trpC*.T-*pyroA* was constructed by an inverse PCR using the primer pair Inverse-2F/Inverse-2R and the plasmid template pUC19-*GFP*-*trpC*.T-*pyrG*. The marker gene *pyroA* was amplified using the primer pair *pyroA*-F/*pyroA*-R and the A6 genomic DNA as template. The two resulting PCR fragments were cyclized to construct pUC19-*GFP*-*trpC*.T-*pyroA*. pUC19-*pyrG*-*catB*.P-*uidA*-*trpC*.T is a plasmid harboring the following sequences at the multiple cloning site: *pyrG*, *catB* gene promoter (*catB*.P), reporter gene *uidA*, and *trpC*.T. *catB*.P and *trpC*.T were cloned from the genomic DNA of *A. nidulans* A6 with the primers *catB*.P-F/*catB*.P-R and *trpC*.T-2F/*trpC*.T-2R, respectively. *uidA* was cloned from *E. coli* genomic DNA with the primers *uidA*-F/*uidA*-R [19]. The resulting DNA fragments, with the linearized pUC19-*pyrG*, were used to construct pUC19-*pyrG*-*catB*.P-*uidA*-*trpC*.T. pUC19-*pyrG*-*prxA*.P-*uidA*-*trpC*.T and pUC19-*pyrG*-*gpdA*.P-*uidA*-*trpC*.T are two plasmids with an arrangement similar to pUC19-*pyrG*-*catB*.P-*uidA*-*trpC*.T except for the promoter components in the inserted DNA fragments. The two plasmids were constructed in the same way.

Plasmids for the generation of *prxA* and *catB*-complemented strains were constructed as follows: The *prxA* expression cassette (*prxA*^com^) and *catB* expression cassette (*catB*^com^) individually encompassing 1.5 kb of 5′-UTR and 1 kb of 3′-UTR of *prxA* and *catB* were amplified by PCR using the genomic DNA of *A. nidulans* A6 and the primer pairs *prxA*^com^-F/*prxA*^com^-R and *catB*^com^-F/*catB*^com^-R, respectively. The linearized pUC19-*pyrG* and *prxA*^com^ as well *catB*^com^ were individually cyclized to construct pUC19-*prxA*^com^-*pyrG* and pUC19-*catB*^com^-*pyrG* using the One Step Cloning Kit.

The D-amino acid oxidase (DAAO) encoding gene of *Rhodotorula gracilis* was synthesized (Tsingke, Shanghai, China) for *A. nidulans* expression, and inserted into pUC19-*pyrG* to construct pUC19-*pyrG*-*gpdA*.P-DAAO-*trpC*.T, in which the transcription of DAAO was controlled via a *gpdA* promoter and a *trpC* terminator. 

### 2.3. Construction of Recombinant Strains

The single guide RNAs (sgRNAs) were synthesized using the GeneArtTM Precision gRNA Synthesis Kit (Invitrogen, Carlsbad, CA, USA). A series of primer pairs, shown in Supplementary Table S4, were designed for the individual gRNA DNA templates assembled by PCR. Table S5 lists the primers designed for recombinant DNA cassette construction.

Δ*catB* and Δ*prxA* were constructed previously [13,14]. To construct the *catB* and *prxA* double disruptant (Δ*catB*Δ*prxA*), the primer pair *catB*-F/*catB*-R was used to amplify the disruption cassette using pUC19-*pyrG* as a PCR template. The resultant *catB* disruption cassette contains the *pyrG* marker gene flanked by 0.1 kb of 5′- and 3′-untranslated region sequences of *catB*. The disruption cassettes of *catB*, together with the corresponding sgRNA and 1 μg Cas9, were transformed into Δ*prxA* to obtain Δ*catB*Δ*prxA*.

pUC19-*prxA*^com^-*pyrG* and pUC19-*catB*^com^-*pyrG* were transformed into Δ*prxA* and Δ*catB* to obtain *prxA* and *catB*-complemented strains (Δ*prxA*-*prxA*^com^, and Δ*catB*-*catB*^com^), respectively. The transformants containing *prxA* and *catB* expression cassettes were confirmed by PCR using the primer pairs *prxA*-1F/*prxA*.T-1R and *catB*-1F/*catB*.T-1R, respectively.

To construct the *catB*-*GFP* strain containing the *catB*-*GFP* expression DNA cassette at the original locus of *catB*, the DNA donor was amplified by PCR using pUC19-*GFP*-*trpC*.T-*pyrG* as a template with the primers *catB*-*GFP*-F/*catB*-*GFP*-R. The PCR product, the corresponding sgRNA, and 1 μg Cas9 were co-transformed into WT to obtain the *catB*-*GFP* strain. The *prxA*-*GFP* and *catB*-*flag* strains were constructed using the same method.

The promoter reporter strains *catB*.P-*uidA* and *prxA*.P-*uidA* were constructed as follows: The expression cassettes for *catB*.P-*uidA* and *prxA*.P-*uidA* were amplified via PCR using the plasmids pUC19-*pyrG*-*catB*.P-*uidA*-*trpC*.T and pUC19-*pyrG*-*prxA*.P-*uidA*-*trpC*.T as templates with the primer pairs *catB*.P-*uidA*-F/*catB*.P-*uidA*-R and *prxA*.P-*uidA*-F/*prxA*.P-*uidA*-R, respectively. sgRNA was designed to insert the *catB*.P-*uidA* and *prxA*.P-*uidA* expression cassettes in the same genomic locus in the gap between *AN9329* and *AN9328*. The expression cassettes, 1 μg Cas9, and the corresponding sgRNAs were transformed into WT to obtain *catB*.P-*uidA* and *prxA*.P-*uidA* promoter reporter strains.

The DAAO expression strain (DAAO/WT) was constructed by transforming pUC19-*pyrG*-*gpdA*.P-DAAO-*trpC*.T into WT. DAAO/Δ*prxA* and DAAO/Δ*catB* were derived from DAAO/WT by deleting *prxA* and *catB* using the same method as used for the construction of Δ*catB* and Δ*prxA*, respectively. The transformants containing DAAO expression cassettes were confirmed via PCR using the primer pair *gpdA*.P-1F/DAAO-1R.

*prxA*.P-*catB*/∆∆, *gpdA*.P-*catB*/∆∆, *gpdA*.P-*catB*/WT, *gpdA*.P-catB*/WT, *gpdA*.P-catB*/∆∆ and *gpdA*.P-catB*-*GFP*/∆∆ were constructed for overexpressing CatB, the putative secretory signal peptide (the first 26 amino acids at the N-terminus) deleted *catB* (catB*) or catB*-*GFP* in WT or in ∆*prxA*∆*catB*. The expression cassette for *prxA*.P-*catB*/∆∆ was amplified via PCR using pUC19-*pyrG*-*prxA*.P-*uidA*-*trpC*.T as a template with the primers *prxA*.P-*catB*-F/*prxA*.P-*catB*-R. The expression cassettes for *gpdA*.P-*catB*/∆∆ and *gpdA*.P-*catB*/WT were amplified using pUC19-*pyrG*-*gpd*A.P-*uidA*-*trpC*.T as a template with the primers *gpdA*.P-*catB*-F/*gpdA*.P-*catB*-R. The plasmid pUC19-*pyrG*-*gpdA*.P-*uidA*-*trpC*.T and the primers *gpdA*.P-catB*-F/*gpdA*.P-catB*-R were used to construct both *gpdA*.P-catB*/WT and *gpdA*.P-catB*/∆∆. The expression cassettes for *gpdA*.P-catB*-*GFP*/∆∆ was amplified using pUC19-*GFP*-*trpC*.T-*pyroA* as a template with the primers *catB*-*GFP*-F/catB*-*GFP*-R. To insert these CatB expression cassettes into the original genomic locus of *catB*, sgRNA was synthesized using the primers *catB*.P-sgF/*catB*.P-sgR. The expression cassettes for *prxA*.P-*catB*/∆∆, *gpdA*.P-*catB*/∆∆ and *gpdA*.P-catB*/∆∆, with the corresponding sgRNAs and Cas9, were transformed into Δ*prxA*. The expression cassettes for *gpdA*.P-*catB*/WT and *gpdA*.P-catB*/WT, with the corresponding sgRNAs and Cas9, were transformed into WT. For *gpdA*.P-catB*-*GFP*/∆∆ construction, the expression cassette was transformed into *gpdA*.P-catB*/∆∆, with Cas9 and the sgRNA which has been used for *catB*-*GFP* strain construction.

*gpdA*.P-*prxA*/∆∆ were constructed for overexpressing *prxA*. The expression cassette was amplified using pUC19-*pyrG*-*gpd*A.P-*uidA*-*trpC*.T as a template with the primers *gpdA*.P-*prxA*-F/*gpdA*.P-*prxA*-R, which was transformed with the corresponding sgRNAs and Cas9 into Δ*catB*.

### 2.4. Confirmation of the Genomic Integration of the Target Genes in the Recombinant Strains

All recombinant strains were confirmed using PCR (Figures S2, S3, S5–S7, S9 and S11). The corresponding primers are listed in Table S6. The copy numbers of the fusion genes *catB*.P-*uidA*, *prxA*.P-*uidA, catB-GFP* and *prxA*-*GFP* integrated in genomic DNA of the individual strains were investigated by Southern blotting as previously described [20,21]. The total genomic DNA of the fungal strains was isolated on 0.8% agarose gel via electrophoresis after digestion with the indicated restriction enzymes (Figures S4 and S8) and subsequently transferred to a Hybond N+ membrane (GE Healthcare, Little Chalfont, UK). Hybridization probes were amplified with the primer sets listed in Table S7 and labeled using digoxigenin (DIG). Hybridization and signal detection were performed using the DIG High Prime DNA Labeling and Detection Starter Kit I (Roche Diagnostics, Basel, Switzerland), according to the manufacturer’s instructions. The presence of a single copy of the integrated target gene was determined based on the estimated sizes of the DNA fragments (Figures S4 and S8).

### 2.5. Conidia Prepared for H_2_O_2_ Resistance Assays

Conidia from the 2-day cultures of each fungus on MM plates were collected in 15 mL sterilized centrifuge tube containing Tween saline (0.1% Tween-80 in 0.8% NaCl). After standing for 15 min, the supernatant conidial suspension was collected and concentrated by centrifugation at 5000 rpm/min for 10 min. The precipitate was resuspended in 2 mL of Tween-saline solution and placed in a hemocytometer (Neubauer chamber) to estimate the conidia concentration. Once the number of conidia had been estimated, they were diluted to the appropriate final concentrations and 10 μL of conidial suspension was plated onto plates containing the indicated concentrations of H_2_O_2_ for resistance testing.

### 2.6. Quantification of Intracellular GFP Levels and β-Glucuronidase (GUS) Activity

Conidia were collected and suspended in Tween-saline solution and the suspension was standardized to a final concentration of about 1 × 10^7^ conidia/mL. A conidia suspension of 100 μL (1 × 10^6^ conidia) was spread on cellophane-coated minimal medium (MM) plates containing 1 mM H_2_O_2_ and incubated for the indicated times. Mycelia were harvested along with cellophane, ground in liquid nitrogen, and resuspended in 500 μL PBS (pH 7.4). The supernatant of the disrupted mycelia was used for fluorescence detection. Protein concentration was determined using the Bradford assay, with bovine serum albumin as the standard. The total protein concentration was diluted to 1 mg/mL, and the GFP fluorescence intensity was measured using a fluorescence spectrophotometer (Hitachi, Tokyo, Japan) with excitation/emission wavelengths of 488/512 nm. For the GUS assay, cell lysates were resuspended in GUS assay buffer (50 mM sodium phosphate buffer pH 7.0, 10 mM β-mercaptoethanol, 10 mM Na_2_EDTA, and 0.1% Triton X-100). After centrifugation, the supernatant was subjected to fluorometric analysis of GUS activity, as described previously [19]. Fluorometric analysis of GUS activity was performed using 4-methylumbelliferyl-b-glucuronide (4-MUG) as a substrate. GUS activity was determined upon the detection of 4-methylumbelliferone (4-MU) fluorochrome generated by the GUS-mediated catalysis of 2 mM 4-MUG hydrolysis using a fluorescence spectrophotometer (HITACHI, Japan) with an excitation wavelength of 365 nm and an emission wavelength of 455 nm. GUS activity was defined as nmol of 4-MU per mg protein per min (abbreviated as mU/mg). 

### 2.7. Quantitative Real-Time PCR

Total RNA and cDNA were extracted and prepared as previously described [14]. Quantitative PCR was performed using the SYBR Green PCR Kit (Toyobo, Osaka, Japan) on a CFX-96 Real-Time PCR system (Bio-Rad, Hercules, CA, USA). The primer pairs RT-catB-F/RT-catB-R and RT-actA-F/RT-actA-R (Table S8) were used to quantify the catB and actA genes, respectively. The relative mRNA levels were normalized to that of the reference gene actA using the 2^−ΔΔCt^ method of relative quantification [14,19]. The experiment was repeated thrice. The mean values ± SD of the three independent experiments were calculated based on one-way analysis of variance with Dunnett’s post hoc test, which were used to identify the statistical differences (* *p* < 0.05; ** *p* < 0.01; and *** *p* < 0.001).

### 2.8. Cell Lysate Catalase Activity Assay

Conidia were spread on cellophane-coated solid plates containing 1 mM H_2_O_2_ and incubated for the indicated times. Fungal cells were collected along with cellophane and ground by liquid nitrogen. The resulted cell debris was dissolved in Catalase Assay Buffer (Beyotime, Shanghai, China) for catalase assay. The crude cell lysate was quantified by the total soluble protein using the Bradford assay. The crude cell lysate containing the protein at the concentration of 0.2 mg/mL was used to determine the enzyme activity using the Catalase Assay Kit (Beyotime, Shanghai, China). This assay is based on colorimetrically measuring the hydrogen peroxide substrate remaining after the action of catalase. The colorimetric method uses a substituted phenol (3,5-dichloro-2-hydroxybenzenesulfonic acid), which couples oxidatively to 4-aminoantipyrine in the presence of hydrogen peroxide and horseradish peroxidase, to give a red quinoneimine dye (N-(4-antipyryl)-3-chloro-5-sulfonatep-benzoquinone-monoimine) that absorbs at 520 nm. One unit of catalase activity was defined as the decomposition of 1 µmol of H_2_O_2_ per minute at pH 7.0 and 25 °C.

### 2.9. Fluorescence Microscopy

Cells were prepared as described previously [13,14]. After cultivation, cells were washed twice with phosphate-buffered saline (PBS; pH 7.4) and observed under a confocal laser scanning microscope (TCS SP8; Leica, Wetzlar, Germany). The fluorescence of the GFP was excited using a 488 nm laser and detected at 510 nm. For imaging endoplasmic reticulum, cells were stained with ER-Tracker Red (Beyotime, Shanghai, China) for 20 min at 37 °C in the dark. The fluorescence of ER-Tracker was excited at 587 nm and emitted at 615 nm.

## 3. Results

### 3.1. PrxA Plays a Role in H_2_O_2_ Resistance in Dormant Conidia

**Figure 1:**
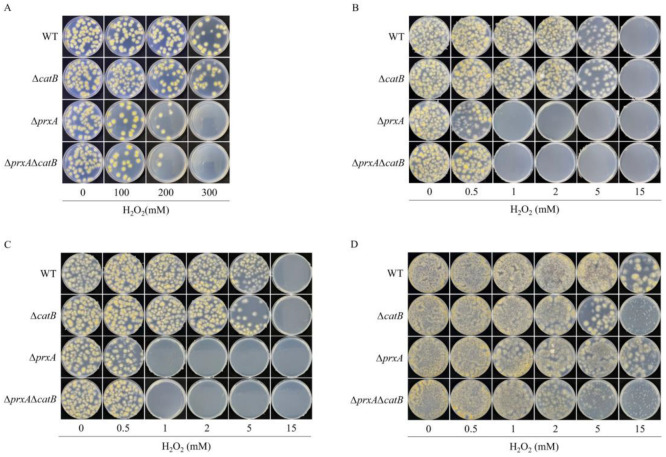
PrxA and CatB defend against different doses of H_2_O_2_ at different growth stages. The strains WT, Δ*catB*, Δ*prxA*, and Δ*prxA*Δ*catB*, at different growth stages, were exposed to the indicated doses of H_2_O_2_ and the resulting colony growth defects were used to determine their H_2_O_2_-sensitivities. (**A**) H_2_O_2_-sensitivities of the dormant conidia. Conidia were suspended in the minimal medium (MM) containing the indicated concentrations of hydrogen peroxide (H_2_O_2_). After treatment for 20 min, 200 conidia of these strains were plated on MM plates and incubated for 2 days at 37 °C. The sensitivity of these strains was evaluated in terms of the colony-forming defects. (**B**) H_2_O_2_-sensitivites of swelling and germinating conidia. (**C**) H_2_O_2_-sensitivities of young hyphae (approximate 6–12 h to old age). (**D**) H_2_O_2_-sensitivities of mature hyphae (24 h-old age). The protocol for the strain treatments shown in (**B**–**D**) is described in detail in the text.

Among the catalase family members, CatA appears to be a unique and indispensable enzyme for protecting conidia against H_2_O_2_ [16]. However, it is unclear whether PrxA contributes to H_2_O_2_ resistance in dormant conidia. Conidia from WT and PrxA gene disruption strain (Δ*prxA*) were treated with a series of H_2_O_2_ concentrations from 0 to 300 mM to investigate the performance of conidial PrxA. Very high concentrations of H_2_O_2_ were used here because dormant conidia are extremely resistant to H_2_O_2_ as described previously, but in fact the environmental H_2_O_2_ generally does not exceed a few millimolar [22,23,24]. After a 20-min exposure, the dormant conidia were plated on MM plates and the colony numbers were used to determine the fungal H_2_O_2_ tolerance. Conidia from Δ*catA* have been reported to be killed by a 20-min exposure of 100 mM H_2_O_2_ [16]. The same treatment also caused adverse effects on the conidial survival of Δ*prxA* (Figure 1A). With the H_2_O_2_ concentrations increasing to 200 mM and 300 mM, the inhibitory effects were more aggravated by Δ*prxA* than WT (Figure 1A). The H_2_O_2_-resistance ability of Δ*prxA* was restored to a level identical to that of WT via *prxA* re-complementation (Figure S1A), indicating that conidial PrxA is involved in the resistance to extremely high H_2_O_2_ concentrations. As a control, *catB* disruption strain (Δ*catB*) and *prxAcatB* double disruption strain (Δ*prxA*Δ*catB*) were constructed (Figure S2). H_2_O_2_ sensitivities of both strains were also investigated, and showed a similar performance to WT and Δ*prxA*, respectively (Figure 1A), excluding conidial CatB in H_2_O_2_ resistance participation, which is in agreement with the experimental results from the previous literature [15,17]. Thus, this study’s results revealed that, besides the previously characterized CatA, PrxA functions as another H_2_O_2_ defender in the dormant conidia.

### 3.2. PrxA and CatB Play Nonredundant Roles in Defense against H_2_O_2_ at Different Growth Stages

Our previous study has reported that Δ*prxA* conidia spotted on MM plates containing H_2_O_2_ exceeding 1 mM could not form surviving colonies [12,14], indicating that PrxA may also protect against H_2_O_2_ in the post dormant conidia stage. CatB activity has been reported to be developmentally regulated, was barely detectable in conidia, and started to accumulate 10 h after conidia inoculation [15,17]. Accordingly, it can be speculated that PrxA and CatB may functionally or cooperatively overlap in protecting fungal cells from H_2_O_2_ damage during the post dormant conidia stage, including conidia swelling and germination, young hyphae development, hyphae maturation, and conidiation. The H_2_O_2_ strains sensitivities lacking either or both PrxA and CatB at different growth stages were investigated to discriminate PrxA and CatB functions during the post dormant conidia stage.

To apply H_2_O_2_ stress to swelling and germinating conidia, we added the dormant conidia into top agar premixed with the indicated concentrations of H_2_O_2_ and plated them onto the precast MM plates to observe the surviving colonies (Figure 1B). Considering the extreme tolerance of dormant conidia to high-dose H_2_O_2_ as shown in Figure 1A and other reports [16], we assumed that this operation could make the conidia encounter a relatively low dose of H_2_O_2_ stress only when swelling and germination occur. After 60 h of cultivation, we found that Δ*prxA* strains could not survive on the plates containing >1 mM H_2_O_2_, whereas Δ*catB* strains showed the same H_2_O_2_ resistance as WT under both low (1 mM) and high (5 mM) stress (Figure 1B), indicating the central role of PrxA, but not CatB, in H_2_O_2_ defense at this stage. Δ*prxA*Δ*catB* strains showed the same H_2_O_2_ sensitivity as Δ*prxA* strains (Figure 1B), further confirming the nonfunctional role of CatB during the conidia swelling and germination stages.

Next, we evaluated the contributions of PrxA and CatB in the growth phase of young hyphae (6–12 h). Conidia were mixed in H_2_O_2_-free top agar and poured on MM plates followed by another layer of top agar containing the indicated doses of H_2_O_2_ (Figure 1C). This operation ensured only the young hyphae just extended from the lower agar in to the upper top agar to be exposed to H_2_O_2_ stress. After 60 h of cultivation, the Δ*prxA* and Δ*prxA*Δ*catB* strains did not form any colonies under a stress higher than 1 mM H_2_O_2_, whereas the Δ*catB* strains were insensitive to 1 mM or 2 mM H_2_O_2_ (Figure 1C), indicating that PrxA was indispensable in protecting young hyphae against H_2_O_2_. Once H_2_O_2_ concentrations exceeding 5 mM, Δ*catB* strains began showing higher sensitivities to H_2_O_2_ than WT (Figure 1C), suggesting the emergence of a functional CatB along in the young hyphae under high H_2_O_2_ stress.

Next, we examined the protection offered by PrxA and CatB in mature hyphae (>18 h). The conidia in H_2_O_2_-free top agar were incubated on MM plates for 24 h, and new H_2_O_2_-containing top agar was poured to provide oxidative stress to the growing hyphae (Figure 1D). After incubating for another 24 h, colonies of the individual strains were compared. The H_2_O_2_ tolerance of all strains was significantly enhanced at this stage compared with the two preceding stages; notably, the WT could even partially survive under 15 mM H_2_O_2_ stress (Figure 1D). Δ*catB* strains exhibited slightly decreased resistance to oxidative stress at 2 mM H_2_O_2_, and the adverse effects became more significant at 5 mM and 15 mM H_2_O_2_, indicating that the role of CatB in H_2_O_2_ resistance increased in significance as the hyphae grew. Re-introduction of *catB* into Δ*catB* (Δ*catB*-*catB*^com^) restored the H_2_O_2_-resistance ability of the mature hyphae of Δ*catB* (Figure S1B), further confirming the functions of CatB in the cells at the mature hypha stage. Interestingly, compared with WT, Δ*prxA* strains did not show more sensitivity to any concentrations of H_2_O_2_ at this stage (Figure 1D), which is contrary to the findings at the earlier growth stages (Figure 1B,C). Therefore, we demonstrated that CatB primarily protected against H_2_O_2_ in mature hyphae, whereas PrxA only played a minor role, especially at H_2_O_2_ concentrations higher than 5 mM. The Δ*prxA*Δ*catB* strains, such as WT, showed robust tolerance to 1 mM H_2_O_2_ stress, and partially survived even at 2 mM and 5 mM H_2_O_2_ (Figure 1D), which is quite different from the sensitivities of the Δ*prxA*Δ*catB* observed during swelling and germinating stages at those concentrations of H_2_O_2_ as shown in Figure 1B,C. Thus, besides PrxA and CatB, an additional, unknown H_2_O_2_ tolerance system must be supporting cellular growth at this stage. Based on the phenotypes of these strains, we concluded that at the growth stage of mature hyphae, both PrxA and CatB contribute to resistance to H_2_O_2_, with CatB playing the leading role.

After the mature hyphae growth stage, conidia will be formed on the specialized aerial hyphae, confounding the hyphae and conidia. In consideration of the difference in performance between the hyphae and the conidia, the cellular H_2_O_2_ tolerance after the mature hyphae stage was no longer characterized.

### 3.3. Expression Profiles of PrxA and CatB Were Correlated with Their H_2_O_2_ Resistance

**Figure 2:**
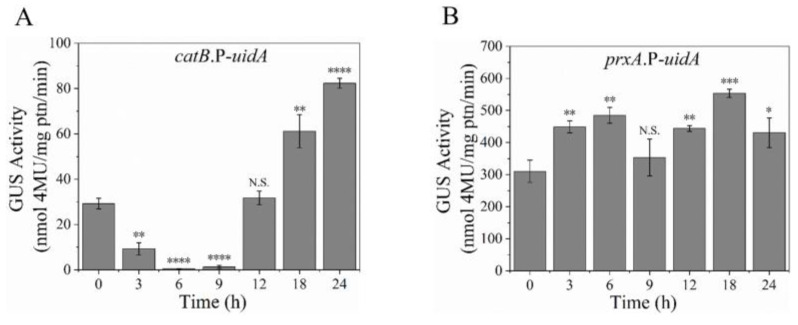
Promoter activity of CatB changed with the growth stage while PrxA was maintained at a high level. (**A**,**B**), Changes in promoter activity accompanying the development of the strains. Promoter activity was indicated by the activity of β-GUS encoded by the *uidA* reporter gene under the control of *catB* promoter (*catB*.P-*uidA*) or *prxA* promoter(*prxA*.P-*uidA*). The conidia of the strains were cultivated at 37 °C on cellophane-coated plates containing 1 mM H_2_O_2_. The strains were collected at the indicated time and disrupted using liquid nitrogen. The cell lysate (1 mg/mL) was used to measure the GUS activity (*n* = 3). Results were presented as mean ± SD and analyzed using one-way ANOVA (N.S., not significant, * *p* < 0.05, ** *p* < 0.01, *** *p* < 0.001, **** *p* < 0.0001).

We predicted that the different intracellular levels of PrxA and CatB may be responsible for their varying contribution to H_2_O_2_ resistance at different stages of cell growth. To verify this, we constructed *catB*.P-*uidA* and *prxA*.P-*uidA* strains in which *uidA*, the β-glucuronidase (GUS) encoding gene [19], was expressed under the promoters of *catB* and *prxA*, respectively (Figure S3). GUS activities of *catB.P*-*uidA* and *prxA.P*-*uidA* strains were used to investigate the promoter activities of both genes during the growth stages from dormant conidia to mature hyphae. The single copy of *uidA* was precisely inserted into the indicated genomic locus of *catB*.P-*uidA* and *prxA*.P-*uidA* strains as confirmed via Southern blotting (Figure S4). As shown in Figure 2A, the cell extracts of H_2_O_2_-induced *catB*.P-*uidA* expression strains exhibited fluctuating GUS activities: they dropped to a minimum at about 6 h, increased to a high level within the following 10 h, and remained stable for the next several hours, which is consistent with the CatB transcription pattern observed in previous literature [17]. Conversely, the *prxA.P*-*uidA* strain extracts showed a relatively constant and high level of GUS activity during all growth stages (Figure 2B). Together with the different H_2_O_2_-resistance abilities of Δ*catB* and Δ*prxA* at different growth stages (Figure 1), it seemed possible that the expression levels of PrxA and CatB directly determined their H_2_O_2_ resistance.

### 3.4. Overexpressing CatB Cannot Enhance H_2_O_2_ Resistance of the Swelling and Germinating Conidia

**Figure 3:**
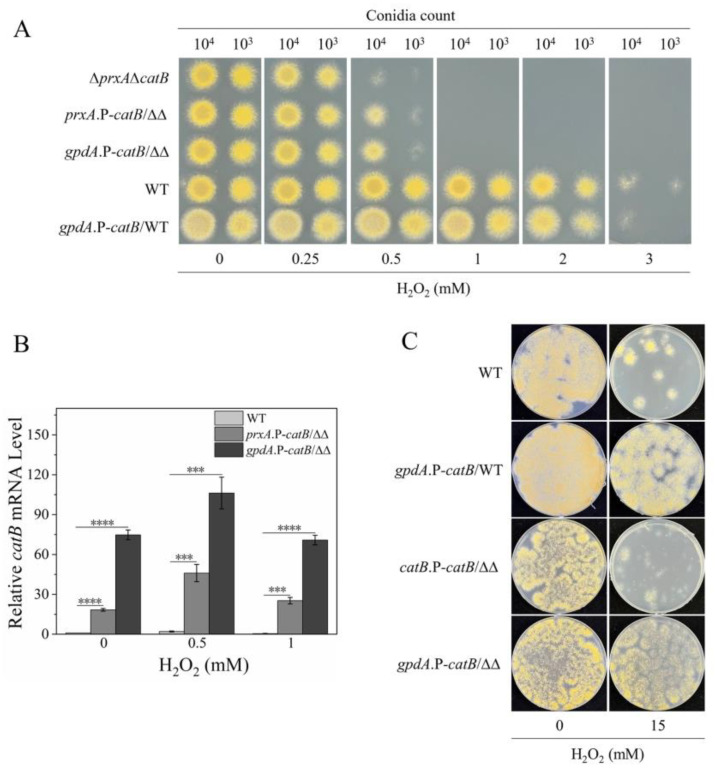
Transgenic overexpression of CatB only conferred higher H_2_O_2_ resistance to strains in the mature hyphae stage. (**A**) Confirmation of the effects of CatB overexpression on the H_2_O_2_ resistance of strains exposed to H_2_O_2_ in the early growth stage. Conidia (1 × 10^4^ and 1 × 10^3^) from various strains were spotted on plates containing the indicated concentrations of H_2_O_2_ for evaluating the H_2_O_2_ sensitivity after 2 days’ cultivation. The strains are shown as follows: ΔΔ, Δ*prxA*Δ*catB*; *prxA*.P-*catB*/∆∆, expressing *catB* by *prxA* promoter in Δ*prxA*Δ*catB*; *gpdA*.P-*catB*/∆∆, expressing *catB* by *gpdA* promoter in Δ*prxA*Δ*catB*; *gpdA*.P-*catB*/WT, expressing *catB* by *gpdA* promoter in WT. (**B**) Relative transcription levels of *catB* in WT, *prxA*.P-*catB*/∆∆, and *gpdA*.P-*catB*/WT under normal and H_2_O_2_ stress conditions. After preculturing conidia on MM plates containing the indicated concentrations of H_2_O_2_ for 6 h, the resulting cells on the cellophane were collected for real-time PCR. The transcription level of *catB* in WT without H_2_O_2_ treatment was set to 1 (mean ± SD; *n* = 3, *** *p* < 0.001, **** *p* < 0.0001, one-way ANOVA). (**C**) Overexpression of CatB conferred resistance to mature mycelia against high concentrations of H_2_O_2_. The newly constructed strain is as follows: *catB*.P-*catB*/∆∆, introducing the *catB* gene under its native promoter into Δ*prxA*Δ*catB*. Conidia of all strains were premixed with top agar without H_2_O_2_ and poured on MM plates. After mycelia were visible (approximate 24 h), new top agar containing the indicated concentrations of H_2_O_2_ was poured to subject the mycelia to oxidative stress. The colonies formed were used to evaluate their H_2_O_2_ resistance.

The above results indicate that the nonfunction of CatB in H_2_O_2_ resistance during the conidia swelling and germination stages would be due to its extremely low expression level, raising an intriguing question: would fungal H_2_O_2_ resistance increase if CatB expression was promoted during these stages? To answer this question, we overexpressed CatB by replacing its native promoter with the stronger and constant *prxA* promoter and investigated the H_2_O_2_ resistance of the swelling and germinating conidia of CatB-overexpression strains by spotting the conidia onto MM plates containing the indicated concentrations of H_2_O_2_ (Figure 3A). Using Δ*prxA*Δ*catB* as the background strain, the *prxA* promoter-dependent CatB expression strain (*prxA*.P-*catB*/ΔΔ) was constructed (Figure S5). Unexpectedly, this strain exhibited only a slightly higher resistance to 0.5 mM H_2_O_2_ than its parent strain Δ*prxA*Δ*catB* (Figure 3A), despite *catB* transcriptional level being markedly enhanced (Figure 3B). Next, we tried to further overexpress CatB by placing the gene under the *gpdA* promoter, a strong constitutive promoter derived from the *A. nidulans gpdA* gene, which encodes glyceraldehyde-3-phosphate dehydrogenase (Figure S5) [25]. The *catB* transcriptional level was further enhanced in the *gpdA*.P-*catB*/ΔΔ strain, irrespective of the presence of H_2_O_2_ (Figure 3B). Nevertheless, as with the *prxA*.P-*catB*/ΔΔ strain, the overexpression of CatB in the *gpdA*.P-*catB*/ΔΔ strain could not compensate for the lack of H_2_O_2_ resistance caused by the absence of PrxA (Figure 3A). Moreover, overexpressing CatB by *gpdA* promoter in WT did not improve the H_2_O_2_ resistance of the strain in its swelling and germinating conidia state (Figure S5 and Figure 3A), clearly indicating that CatB does not protect the swelling and germinating conidia against H_2_O_2_. To investigate whether CatB functionalization was associated with the growth phase, we compared the H_2_O_2_-tolerance of the CatB-overexpression strain and WT in their mature hyphae stage (Figure 3C). We observed that both WT and Δ*prxA*Δ*catB* mutants, under 15 mM H_2_O_2_ stress, survived significantly better when CatB was overexpressed (Figure 3C), confirming that CatB functionalization confined to the mature hyphae stage.

### 3.5. Extracellular Secretion of CatB Is Necessary for H_2_O_2_ Resistance

**Figure 4:**
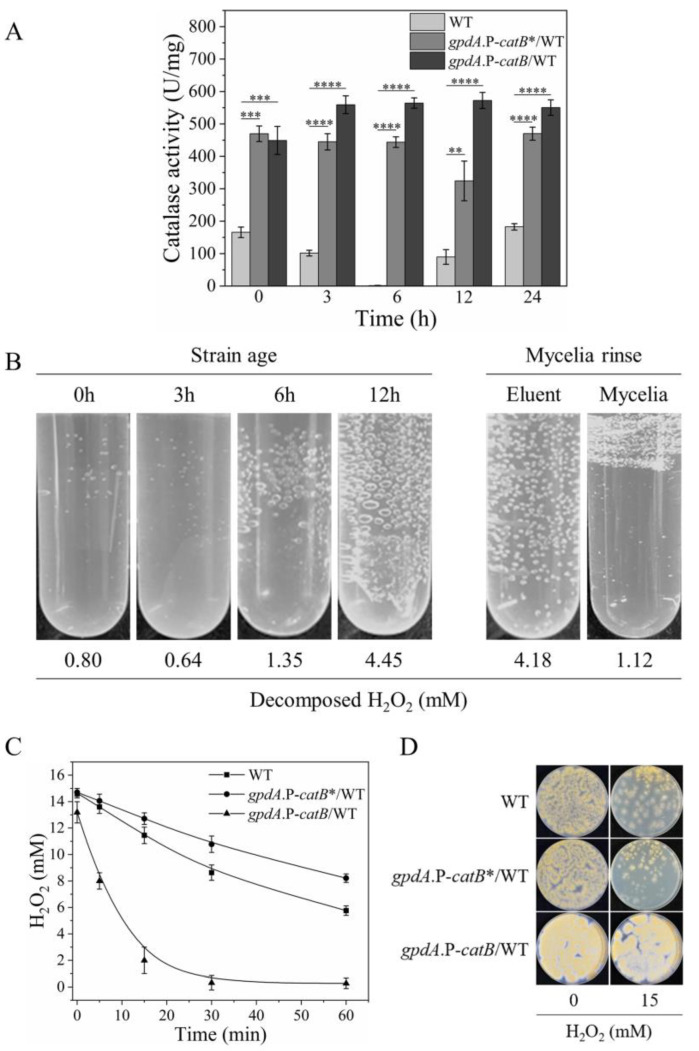
Growth stage-dependent secretion of CatB is needed for H_2_O_2_ resistance. (**A**) Comparison of H_2_O_2_ decomposition activities of the crude cell lysates from WT and CatB-overexpressing strains at different growth stages. Cell lysates were derived from the fungal cells cultivated on MM plates containing 1 mM H_2_O_2_ covered by cellophane for the indicated times (mean ± SD; *n* = 3, ** *p* < 0.01, *** *p* < 0.001, **** *p* < 0.0001, One-way ANOVA). (**B**) H_2_O_2_ decomposition by intact cells from different growth stages. Different gas bubbles produced by the intact CatB-overexpressing strains in the early and late growth stages. Approximately 1 cm^2^ cellophane covered with *gpdA*.P-*catB*/WT cells were cut from the plates cultivated for the indicated times and immersed in 10 mL PBS solution containing 15 mM H_2_O_2_ for 5 min. Left panel, gas bubbles produced by these fungal cells. Right panel, 12 h cultivated fungal cells were rinsed, and the resulting mycelia and the eluent were compared for their catalase activities. The decreased concentrations of H_2_O_2_ calculated by the absorbance decreases at 240 nm are shown at the bottom of each sample. (**C**) Overexpression of the truncated CatB lacking the N-terminal signal sequence did not accelerate H_2_O_2_ decomposition. Mature mycelia (12 h cultivation) of three strains from 1 cm^2^ cellophane were immersed in 10 mL PBS containing 15 mM H_2_O_2_ to compare the H_2_O_2_ decomposition rates. H_2_O_2_ decomposition was monitored by detecting the absorbance changes at 240 nm. Error bars (SD) were calculated from biological triplicates. (**D**) H_2_O_2_ resistance needs the secreted CatB. After 24 h cultivation of the indicated strains, 15 mM H_2_O_2_-containing top agar was covered on the plates to compare the H_2_O_2_ resistance of these strains. *gpdA*.P-*catB*/WT is the WT CatB overexpression strain and *gpdA*.P-catB*/WT is the deduced N-terminal signal peptide (26 amino acids)-truncated CatB overexpressing strain.

Next, we investigated the reasons underlying the distinct outcomes of overexpressing CatB during the different growth stages. We first tested whether CatB exhibited different activities at the different growth stages by comparing the total catalase activities in the crude cell lysates of CatB-overexpression strains cultivated on H_2_O_2_-containing MM plates for 0, 3, 6, 12, and 24 h. As a control, catalase activity in WT strains was also measured. As shown in Figure 4A, the level of catalase activity in WT between 0 and 24 h had a special change of descend first then ascend, consistent with the promoter activity change of CatB during this time period (Figure 2A). However, in cell lysates of CatB-overexpression strains, the catalase activities were relatively constant and far higher than in the WT cells at any growth stages (Figure 4A). These results suggest that the activity change of CatB was not responsible for its distinct contribution to H_2_O_2_ resistance during different growth stages.

We observed that bubbles were produced upon dipping *gpdA*.P-*catB*/WT cells of different growth stages in water containing 15 mM H_2_O_2_ (Figure 4B). The bubbles should be due to the rapidly released O_2_ derived from the decomposition of H_2_O_2_ catalyzed by catalase, which can be monitored and calculated by the absorbance decreases at 240 nm (Figure 4B, on the bottom). Interestingly, many more bubbles were produced by the hyphae of 12-h strain age than the cells of 3-h strain age (Figure 4B), though the total catalase activities of the two cells’ lysate showed the same levels (Figure 4A). Thus, more O_2_ bubbles may suggest that more CatB was secreted extracellularly in these cells. Rinsing the 12-h strain age mycelia in phosphate-buffered saline ended the rapid decomposition of H_2_O_2_, whereas the eluent showed high catalase activity (Figure 4B, on the bottom). Based on these observations, we deduced that the functional catalase would be distributed on the cell surface and linked to the cells via noncovalent bonds. To verify this, we constructed a strain overexpressing CatB*, which is a truncated CatB lacking the putative secretory signal peptide (the first 26 amino acids at the N-terminus) (Figure S6) [26,27]. As predicted, the mature hyphae of the truncated CatB overexpressing strains decomposed H_2_O_2_ only to the extent of WT strains, losing the high catalase activity (Figure 4C). In accordance, overexpressing the truncated CatB did not promote the fungal H_2_O_2_ resistance (Figure 4D). These results emphasize that CatB must be secreted for its functionalization and also imply that CatB secretion is confined in the late growth stage.

### 3.6. Functionalized PrxA and CatB Localize in Cytosol and Cell Surface, Respectively

**Figure 5:**
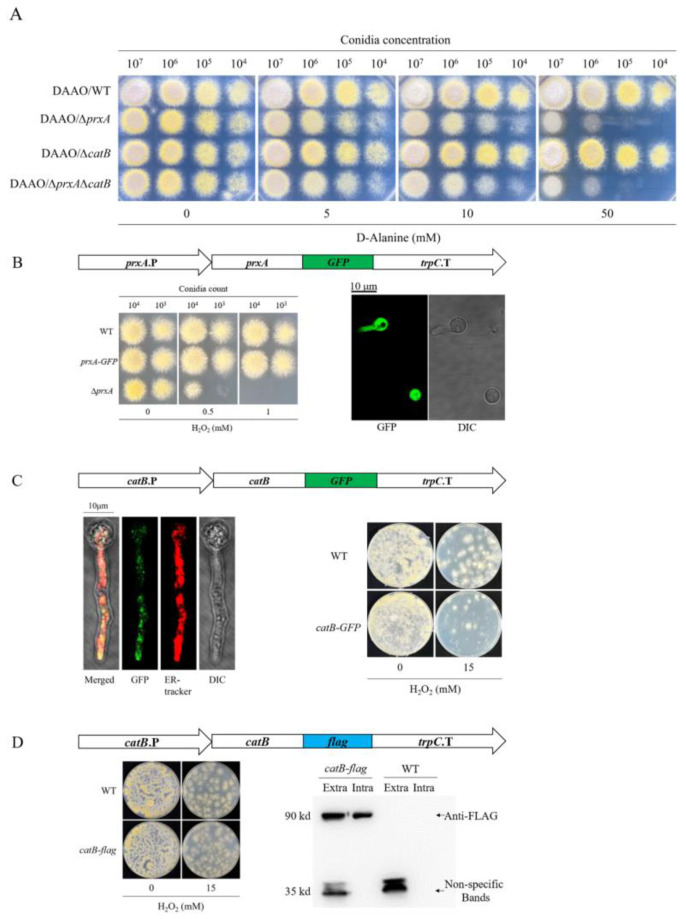
Functional PrxA and CatB localize in the cytosol and on the cell surface, respectively. (**A**) Sensitivities of cells to the intracellular H_2_O_2_ produced by the D-amino acid oxidase (DAAO) expression system. *Rhodotorula gracilis* DAAO was expressed in WT to construct the DAAO/WT strain. DAAO/Δ*prxA*, DAAO/Δ*catB*, and DAAO/Δ*prxA*Δ*catB* were derived from DAAO/WT by deleting the corresponding genes. Conidia from various strains were spotted on plates containing the indicated concentrations of D-alanine for evaluating the H_2_O_2_ sensitivity after 2 days of cultivation. (**B**) The H_2_O_2_ resistance and cytosol location of PrxA-GFP in the swelling and geminating cells. C-terminal GFP-tagged PrxA (PrxA-GFP) was expressed using the *prxA* promoter and *trpC* terminator in Δ*prxA* (top panel). To confirming the normal functioning of PrxA-GFP, conidia from WT, Δ*prxA* and *prxA*-*GFP* were spotted on the MM plates containing the indicated concentrations of H_2_O_2_ for the H_2_O_2_ resistance assays. After 5 h cultivation of the conidia of *prxA*-*GFP*, the fluorescence of PrxA-GFP in the swelling and germinating conidia were analyzed via fluorescence microscopy. (**C**) C-terminal fusion of CatB with GFP led to an endoplasmic reticulum (ER) location of CatB-GFP, and H_2_O_2_-resistant impairment of the mature fungal hyphae. CatB-GFP expression cassette is shown in the top panel. Mature fungal hyphae (24 h) of *catB*-*GFP* were stained red by ER-Tracker for their visualization via fluorescence microscopy. 24 h cultivated WT and *catB*-*GFP* were covered with top agar containing 0 or 15 mM H_2_O_2_ for comparing the H_2_O_2_ resistance. (**D**) FLAG tag did not affect the function of CatB in H_2_O_2_ resistance and indicated CatB localizing on the cell surface of mature hyphae. CatB-FLAG expression cassette was showed in the top panel. 24 h cultivated WT and *catB*-*flag* were covered with top agar containing 0 or 15 mM H_2_O_2_ for comparing the H_2_O_2_ resistance. The intracellular residual CatB-FLAG and the extracellularly secreted CatB-FLAG were confirmed by western blotting using an anti-FLAG monoclonal antibody. The mycelia from a plate were collected and well-rinsed with PBS. The eluent was centrifuged to 1 mL and 10 μL of the eluent was loaded for analysis. The rinsed mycelia were disrupted by liquid nitrogen and the cell extract was modulated up to 1 mL, of which 10 μL was loaded for analysis. The samples from WT were used to test the specificity of the anti-FLAG antibody.

Based on the above results, we speculated that PrxA and CatB defend against H_2_O_2_ intracellularly and extracellularly, respectively. To verify this, the sensitivities of Δ*prxA* and Δ*catB* to endogenous H_2_O_2_ were investigated. DAAO catalyzes the conversion of D-amino acids to their corresponding alpha-keto acids, producing H_2_O_2_ in the process. DAAO from the yeast *R. gracilis* has been successfully applied to specifically generate endogenous H_2_O_2_ in mammalian cells [28,29]. Here, we attempted to use this system in *A. nidulans* for the generation of endogenous H_2_O_2_. Expression of DAAO in WT (DAAO/WT) and Δ*catB* (DAAO/Δ*catB*) had no influence on the growth of the spotted conidia of both strains exposed to various concentrations of D-Ala (Figure 5A). In contrast, growth inhibition was detected on the DAAO-expressing Δ*prxA* (DAAO/Δ*prxA*) and Δ*prxA*Δ*catB* (DAAO/Δ*prxA*Δ*catB*) along with the increase in D-Ala concentrations in the media, confirming our speculation that PrxA rather than CatB counters the production of endogenous H_2_O_2_ (Figure 5A). Next, we investigated the cellular locations of the functionalized PrxA and CatB by the newly constructed GFP-tagged PrxA and CatB expression strains (*prxA*-*GFP* and *catB*-*GFP*) (Figure S7). The single copy of *GFP* was precisely inserted into the indicated genomic locus of *prxA*-*GFP* and *catB*-*GFP* strains as confirmed via Southern blotting (Figure S8). The fluorescence of PrxA-GFP diffused through all cells of the swelling and germinating conidia (Figure 5B), indicating that PrxA localizes to cytosol. The fluorescence of CatB-GFP in the mature hyphae was distributed as discontinuous spots (Figure 5C), indicating that it should localize to specific organelles. CatB-GFP partially coincided with the dyed endoplasmic reticulum (ER), indicating its ER retention (Figure 5C). However, no fluorescence was observed at the surface of hyphae, suggesting that CatB-GFP was not secreted to the cell wall, which does not agree with the predicted location of functional CatB. We speculated that the GFP tag may sterically hinder the secretion of CatB-GFP, leading to its accumulation in the ER. The CatB-GFP expression strains could not survive as did WT on plates containing 15 mM H_2_O_2_ (Figure 5C), further supporting our speculation. Contrary to CatB-GFP, the cytosol should be the right location of PrxA-GFP, since cytosolic PrxA-GFP defended against H_2_O_2_ just as PrxA did (Figure 5B).

To confirm the secretion and cell surface immobilization of CatB, we replaced the C-terminal GFP with a FLAG tag (Figure S7), which was expected to not impede CatB secretion and also make the tagged protein detectable via western blotting. As shown in Figure 5D, CatB-FLAG and CatB similarly protected mature hyphae against 15 mM H_2_O_2_, suggesting the extracellular secretion of CatB-FLAG. To directly detect the secreted CatB-FLAG on the cell surface, the mature hyphae were rinsed, and the washed eluate was subjected to western blotting using an anti-FLAG antibody. As expected, CatB-FLAG was detected in the washed eluate (Figure 5D). The cell lysate from the rinsed cells was also analyzed. We found that the cell lysate had slightly lower levels of CatB-FLAG than the same volume of washed eluate (Figure 5D), indicating that more CatB was secreted.

### 3.7. Cytosolic Localized CatB Cannot Functionally Substitute PrxA at Germinating Conidia Stage 

As shown in Figure 3A, CatB’s intracellular abundance in *gpdA*.P-*catB*/ΔΔ did not show any improved antioxidant ability comparing to its parent strain Δ*prxA*Δ*catB* (ΔΔ) at the swelling and germinating conidia stages, leading us to hypothesize the specific CatB organelle-location may be a barrier for its antioxidant function. To verify that, we attempted translocating CatB from ER to cytosol to observe fungal resistance changes to H_2_O_2_. We overexpressed a GFP-fused N-terminal signaling peptide absent CatB (CatB*-GFP) in Δ*prxA*Δ*catB* to construct the strain *gpdA*.P-catB*-*GFP*/ΔΔ (Figure S9). Compared with the WT strain expressing GFP-tagged CatB (CatB-GFP), the fluorescence in the swelling and germinating conidia of *gpdA*.P-catB*-*GFP*/ΔΔ strain was greatly enhanced and diffused in the whole fungal cell (Figure S10A), indicating the truncated CatB was successfully overexpressed in the germinating conidia’s cytosol. However, CatB*-GFP expression strain H_2_O_2_ tolerance was similar to the signal peptides retaining CatB-overexpression strains and was far inferior to that of the PrxA-overexpression strains (*gpdA*.P-*prxA*-*GFP*/ΔΔ) (Figure S10B,C), which was constructed by replacing *prxA* promoter with *gpdA* promoter in Δ*catB* (Figure S11). These results suggested that the reason CatB cannot substitute PrxA for H_2_O_2_ resistance is not due to their different cellular locations.

## 4. Discussion

Both Prxs and catalases possess H_2_O_2_-decomposing activities. However, the exact reason underlying the existence of two functionally duplicated systems in one organism remains unclear. Our present study successfully discriminated the antioxidant functions of *A. nidulans* PrxA and CatB. During the early growth stages, including conidial swelling, germination, and the young hyphae, the fungus can only resist low concentrations of H_2_O_2_ and PrxA acts as an indispensable antioxidant in this period. The mature hyphae can resist high concentrations of H_2_O_2_, mainly due to CatB secreted and immobilized on the cell wall. We verified CatB secretion is confined at the mature hyphae growth stage. Therefore, the antioxidant network in the model fungus *A. nidulans* involves an asynchronous collaboration between Prx and catalase to defend against H_2_O_2_.

The abundant expression of CatB is highly correlated with its protective function under H_2_O_2_ stress. However, fungal H_2_O_2_ resistance is not determined by CatB expression levels exclusively, because constant high expression of CatB conferred high H_2_O_2_ resistance upon mature hyphae but not young hyphae (Figure 3A,C). Furthermore, our results suggest that the rapid decomposition of H_2_O_2_ around the mature hyphae by the secreted CatB relieved the oxidative stress, and this secretion ability may be lacking in younger cells (Figure 4B). The timing of CatB secretion seems to be important for its function, which must be tightly regulated. Protein secretion is a complicated and energy-consuming process that involves vesicle biogenesis, cargo loading, concentration and processing, vesicle transport and targeting, vesicle docking, and Ca^2+^-dependent vesicular fusion with the plasma membrane [30,31]. The forced secretion of CatB during the early growth phase might exhaust energy and occupy the secretory routes of other essential proteins involved in nutrition intake or cell wall synthesis, eventually leading to an intracellular traffic jam of protein cargo. Since the cellular machinery for CatB secretion is not fully equipped, suppressing CatB expression during the early growth stage might be a behavior favored by natural selection for *A. nidulans*.

The Prx and catalase of *S. pombe* have also been intensively investigated and functionally discriminated for their roles in H_2_O_2_ scavenging. The yeast Prx Tpx1 defends against low levels of intracellular H_2_O_2_ produced during aerobic growth, whereas the yeast catalase chiefly scavenges extracellular H_2_O_2_ [10,32]. By contrast, *A. nidulans* Δ*prxA* does not show O_2_ sensitivity and both PrxA and CatB are required to defend against exogenous H_2_O_2_. Another notable antioxidant system distinction between *A. nidulans* and *S. pombe* is the different activation periods of their individual catalase. The *A. nidulans* CatB activation is confined in the mature hyphae growth stage; whereas the *S. pombe* catalase may stay active in the early growth stages, since catalase-deficient *S. pombe* seeded on the plate containing 1 mM H_2_O_2_ were nonviable [10]. The close resemblance between the *A. nidulans* and *S. pombe* antioxidant systems is that the catalases’ overexpression cannot suppress the growth defects caused by the absences of their individual Prx under the exogenous H_2_O_2_ stress conditions (Figure S2B) [10]. Prxs’ nonsubstitutability by catalases in H_2_O_2_ tolerance in the two fungal species suggested the other functions of Prx, such as molecular chaperoning and signaling, may also be indispensable during the antioxidant process.

We observed an interesting phenomenon when we compared the phenotypes of Δ*prxA*Δ*catB* strains at different growth stages: 1 mM H_2_O_2_ was lethal to cells in the early stage but did not exert any stress on the late-growth hyphae, which could even partially survive 5 mM H_2_O_2_ (Figure 1B–D). This suggests the possibility of other important H_2_O_2_-resistant systems that are activated only at the later growth stage. These unknown systems may include the other previously reported multiple peroxidases and catalases. Although single and multiple gene mutants of these enzymes had no effect on their function in the early growth stage [33], they may function in the late growth stage, especially cooperatively. The distinct cellular permeability to H_2_O_2_ exhibited by hyphae in the two growth stages may also result in their different H_2_O_2_ resistance. H_2_O_2_ does not freely diffuse into human, *E. coli*, and *S. cerevisiae* cells; it is a regulated biological process [9,34,35,36]. In *S. cerevisiae*, limiting H_2_O_2_ diffusion by actively decreasing the cellular permeability is an important protective strategy against extracellular H_2_O_2_ [35,37]. The cellular permeability to H_2_O_2_ is five-fold lower in the stationary phase than in the exponential phase. Consequently, when challenged with H_2_O_2_, more *S. cerevisiae* cells in the stationary phase survive than those in the exponential phase, similar to *A. nidulans*. The cell wall and plasma membrane of yeast constitute the permeability barriers to H_2_O_2_ [35,36], which may be common for fungi due to their similar cell structures. Future studies should focus on identifying the key components contributing to the barrier function against H_2_O_2_ as well as the molecular mechanisms by which these factors are regulated in different growth phases.

## 5. Conclusions

The major peroxiredoxin (PrxA) and the key catalase (CatB) of *Aspergillus nidulans* play nonredundant roles in H_2_O_2_ resistance. PrxA is indispensable for protecting against H_2_O_2_-induced damage in the conidia or young hyphae but not in mature hyphae. By contrast, CatB only performs an antioxidant role in mature hyphae. Functionalization of CatB requires it to be secreted and immobilized on the cell wall, both of which are confined to the mature hyphae stage. Therefore, fungal catalase and peroxiredoxin have a distinct division of labor in counteracting H_2_O_2_.

## Data Availability

All data are contained within the article and Supplementary Materials.

## Supplementary Materials

The following supporting information can be downloaded at: https://www.mdpi.com/article/10.3390/antiox12071333/s1. Figure S1: Re-introduction of *prxA* and *catB* to their gene-deletion strains recovered their H_2_O_2_-resstance abilities identical to WT level; Figure S2: Disruption of *catB* gene in Δ*prxA*; Figure S3: Construction of the promoter reporter strains of *A. nidulans*; Figure S4: Identification by Southern blotting analyses of *A. nidulans* transformants carrying the *catB*.P-*uidA* or *prxA*.P-*uidA* genotype; Figure S5: Construction of promoter substitution strains in *A. nidulans*; Figure S6: Construction of overexpression strains lacking signal peptides in *A. nidulans*; Figure S7: Construction of *catB*-*GFP*, *catB*-*flag*, and *prxA*-*GFP* expression strains of *A. nidulans*; Figure S8: Identification by Southern blotting analyses of *A. nidulans* transformants carrying the *catB*-*GFP* or *prxA*-*GFP* genotype; Figure S9: Construction of the strains overexpressing CatB* or CatB*-GFP in Δ*prxA*Δ*catB*; Figure S10: Cytosolic CatB cannot functionally substitute PrxA at the swelling and germinating conidia stage; Figure S11: Construction of *prxA* promoter was replaced by *gpdA* promoter strains in *A. nidulans*; Table S1: Strains used in this study; Table S2: Plasmids used in this study; Table S3: Primers used for plasmids construction; Table S4: Primers used for sgRNA synthesis; Table S5: Primers used for strains construction; Table S6: Primers used for strains verification; Table S7: Primers used for probe synthesis; Table S8: Primers used for Q-RT-PCR.
