# Supplementary material for: Cytosol Peroxiredoxin and Cell Surface Catalase Differentially Respond to H2O2 Stress in Aspergillus nidulans
Source: Antioxidants (Basel). 2023 Jun 23;12(7):1333. doi: 10.3390/antiox12071333 (PMC10376852; doi:10.3390/antiox12071333)
Supplement: Supplementary file 1 [file antioxidants-12-01333-s001.zip › Supplementary figure_Final version.pdf]

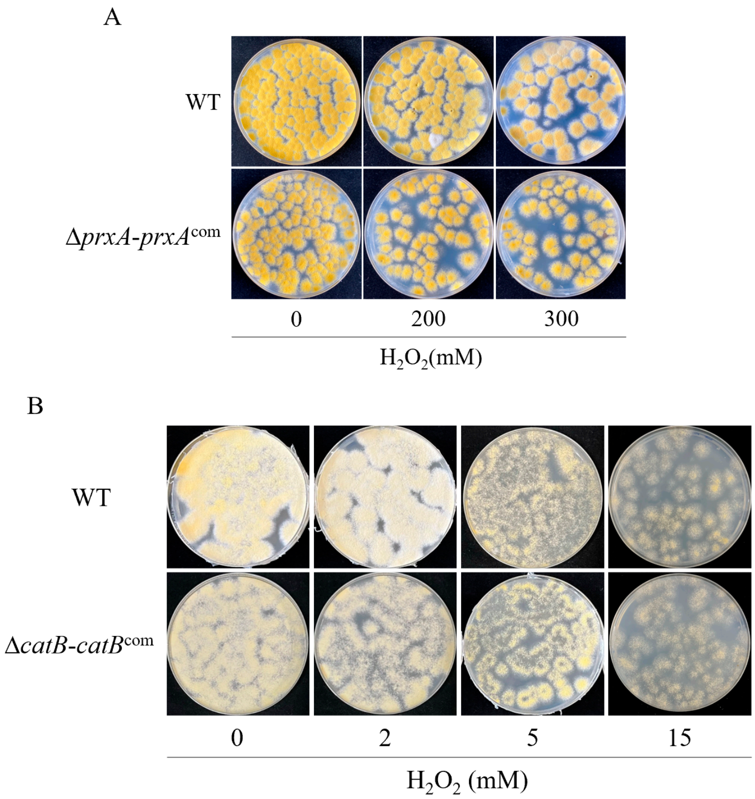

Figure S1. Re-introduction of *prxA* and *catB* to their gene-deletion strains recovered their H_2O_2-resstance abilities identical to WT level. A, H_2O_2-sensitivities of the dormant conidia. Conidia were suspended in the minimal medium (MM) containing the indicated concentrations of H_2O_2 for 20 min, and then plated on MM plates and incubated for 2 days at 37°C. B, H_2O_2-sensitivities of mature hyphae (24 h-old age). The operation is similar to that described in Figure 1D.

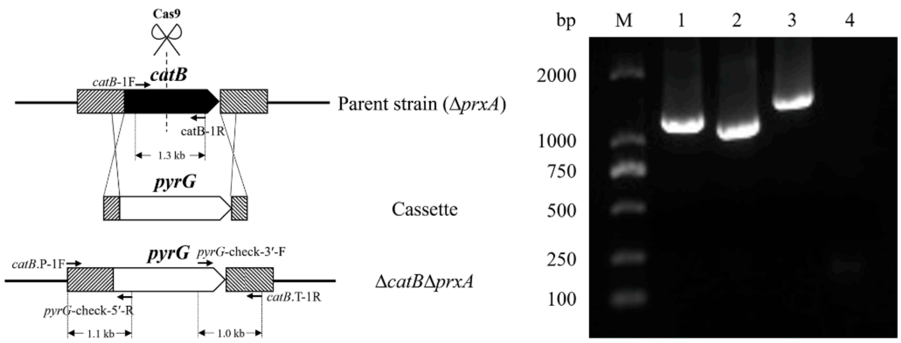

Figure S2. Disruption of *catB* gene in Δ*prxA*. *catB* gene was replaced by the marker gene *pyrG* via the homology-directed repair pathway, assisted by the CRISPR/Cas9 system (left). PCR analysed to verify the isolated disruptant (right). Replacement of *catB* by *pyrG* in the *catA*-disruptant was verified using the primer pairs *catB*.P-1F/*pyrG*-check-5′-R (lane 1), *pyrG*-check-3′-F/*catB*.T-1R (lane 2). Deletion of the *catB* gene in the parent strain (lane 3) and the disruptant strain (lane 4) were confirmed using the primers *catB*-1F/*catB*-1R. The corresponding primers were listed in Supplementary Table 6.

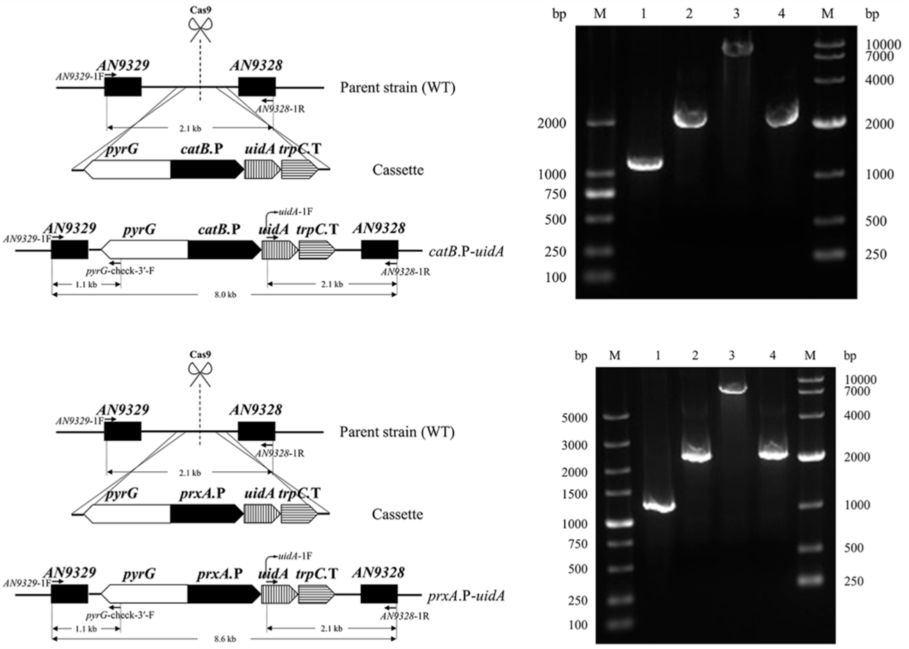

Figure S3. Construction of the promoter reporter strains of *A. nidulans*. Strategy for constructing the *catB*.P-*uidA* and *prxA*.P-*uidA* strains (left) and the validation of the resulting transformants (right). The *catB*.P-*uidA* expression strain was verified with the primer pairs *AN9329*-1F/*pyrG*-check-3′-F (lane 1) and *uidA*-1F/*AN9328*-1R (lane 2). The primer pair *AN9329*-1F/*AN9328*-1R was used to amplify the corresponding regions in the isolated transformant (lane 3) and parent (lane 4) strains. The strain *prxA*.P-*uidA* was verified in the same way as the indicated primers. The corresponding primers were listed in Supplementary Table 6.

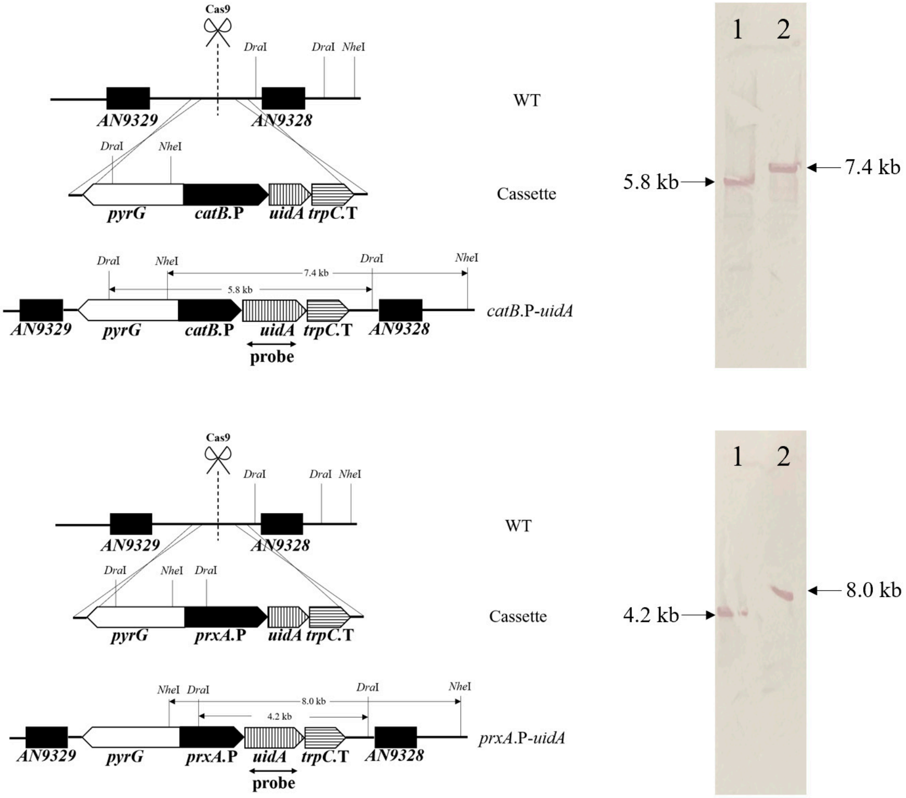

Figure S4. Identification by Southern blotting analyses of *A. nidulans* transformants carrying the *catB*.P-*uidA* or *prxA*.P-*uidA* genotype. Strategy of the cleavage site and probes was shown on the left, and the verification results were shown on the right. The binding results of *Dra*I or *Nhe*I digested fragments of the transformants genomes with *uidA* probe were shown in lane 1 and lane 2, respectively. Primers for the *uidA* probe were listed in Supplementary Table 7.

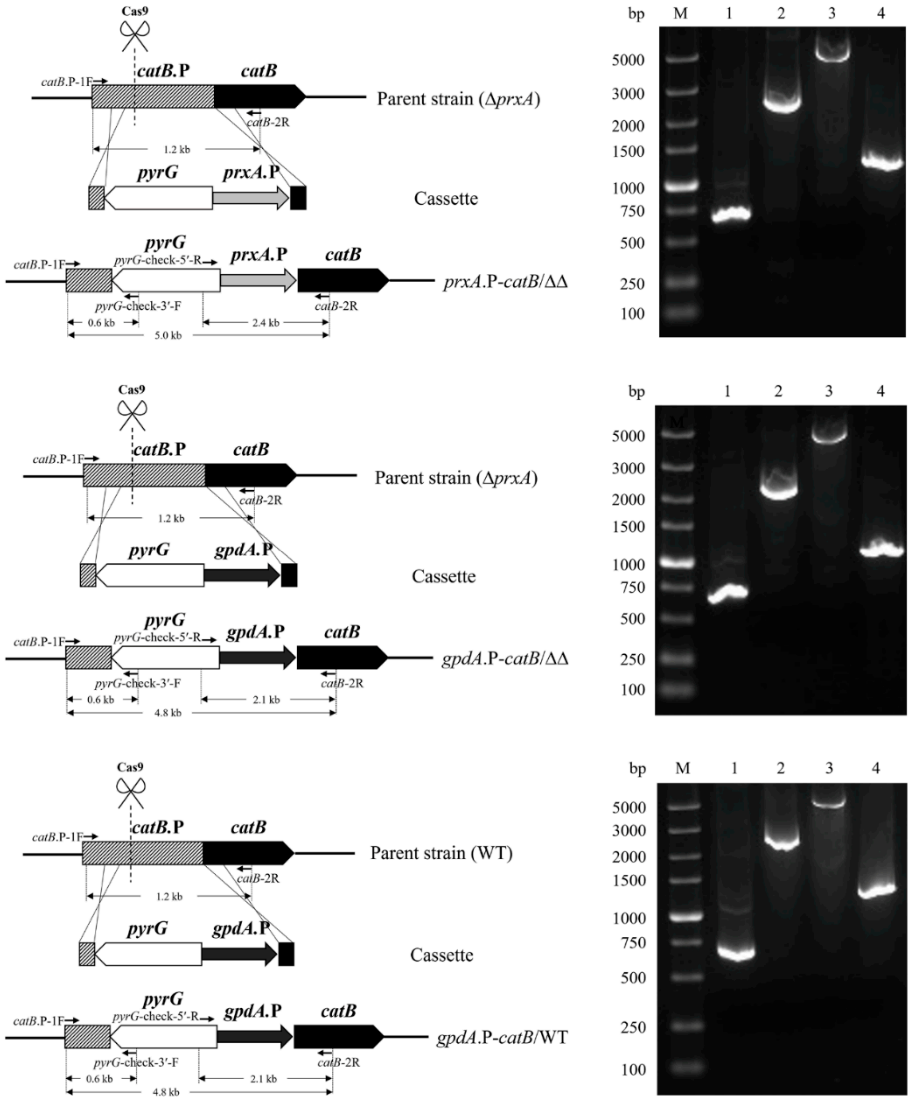

Figure S5. Construction of promoter substitution strains in *A. nidulans*. Promoter replacement strategies for homologous recombinant strains *prxA*.P-*catB*/ΔΔ, *gpdA*.P-*catB*/ΔΔ, and *gpdA*.P-*catB*/WT were shown on the left. Validation of the corresponding recombination by PCR with the indicated primers was shown on the right. The *prxA*.P-*catB*/ΔΔ was verified with the primer pairs *catB*.P-1F/*pyrG*-check-3′-F (lane 1) and *catB*-2R/*pyrG*-check-5′-R (lane 2). The primer pair *catB*.P-1F/*catB*-2R was used to amplify the corresponding regions in the isolated transformant (lane 3) and parent (lane 4) strains. Similar methods were used to validate the isolated transformants of *gpdA*.P-*catB*/ΔΔ and *gpdA*.P-*catB*/WT. The corresponding primers were listed in Supplementary Table 6.

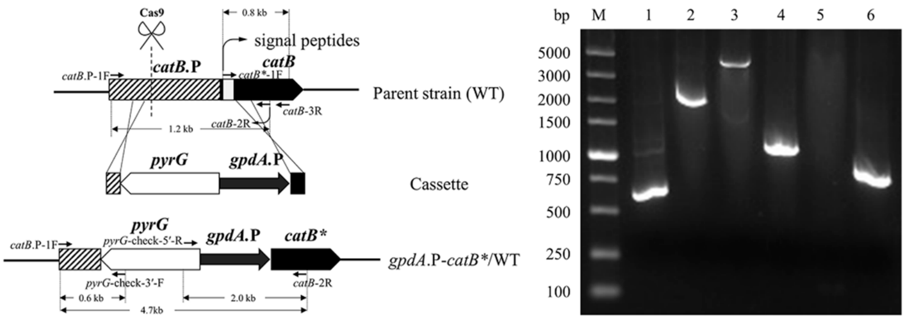

Figure S6. Construction of overexpression strains lacking signal peptides in *A. nidulans*. The strategy to construct the strain expressing truncated CatB under the promoter of *gpdA*.P (left) and the validation of the resulting transformants (right). The *gpdA*.P-*catB**/WT was verified with the primer pairs *catB*.P-1F/*pyrG*-check-3′-F (lane 1), *catB*-2R/*pyrG*-check-5′-R (lane 2). The primer pair *catB*.P-1F/*catB*-2R was used to amplify and discriminate the corresponding region in the isolated transformant (lane 3) and parent (lane 4) strains. The primer pair *catB**-1F/*catB*-3R was used to amplify and discriminate the corresponding region in the isolated transformant (lane 5) and parent (lane 6) strains. The corresponding primers were listed in Supplementary Table 6.

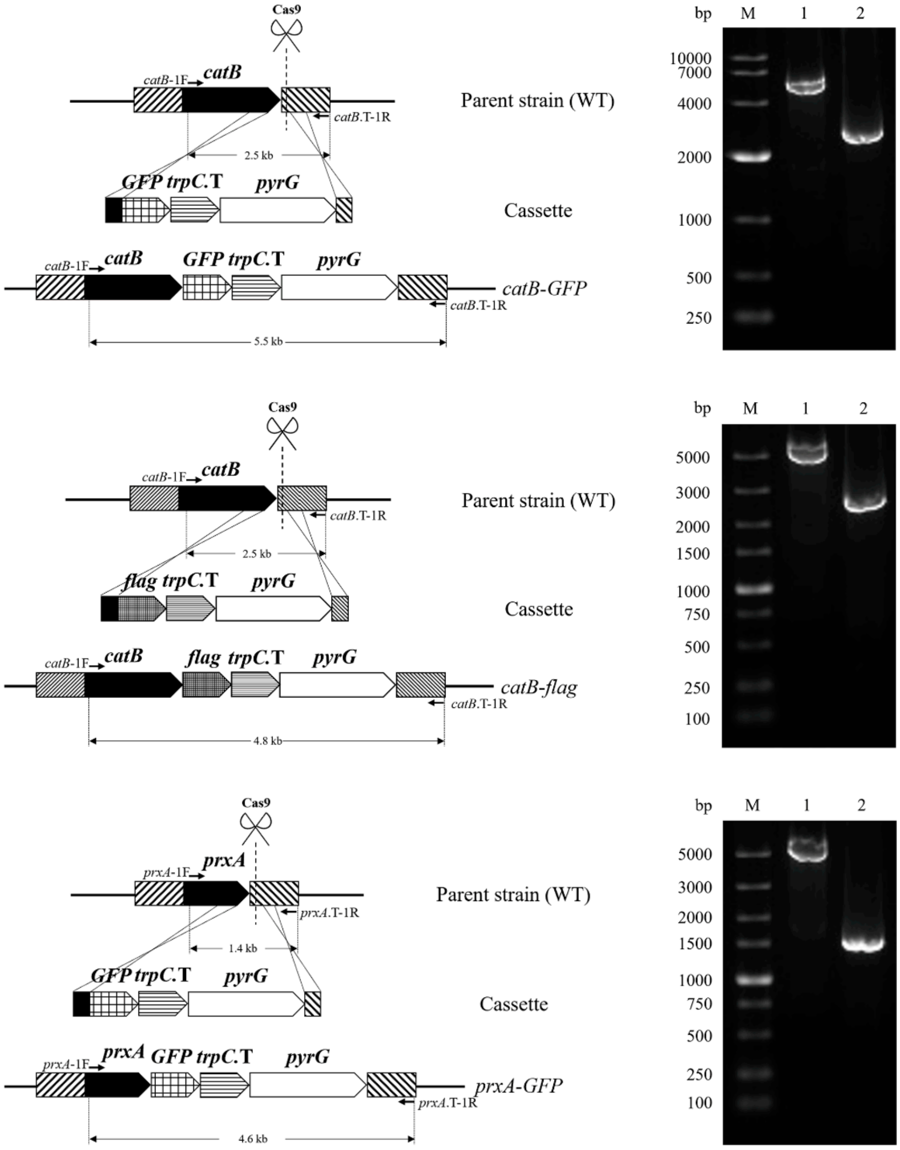

Figure S7. Construction of *catB-GFP*, *catB-flag*, and *prxA-GFP* expression strains of *A. nidulans*. Left, schematic diagrams of the replacement of the target genes by the *GFP* or *flag*-fused target genes via the homology-directed repair pathway, assisted by the CRISPR/Cas9 system. Right, PCR confirmations of the resulting recombinant strains. To verify the correct recombinant strains, the indicated primer pairs were used to amplify the corresponding region in the isolated transformant (lane 1) and the parent (lane 2) strains. The corresponding primers were listed in Supplementary Table 6.

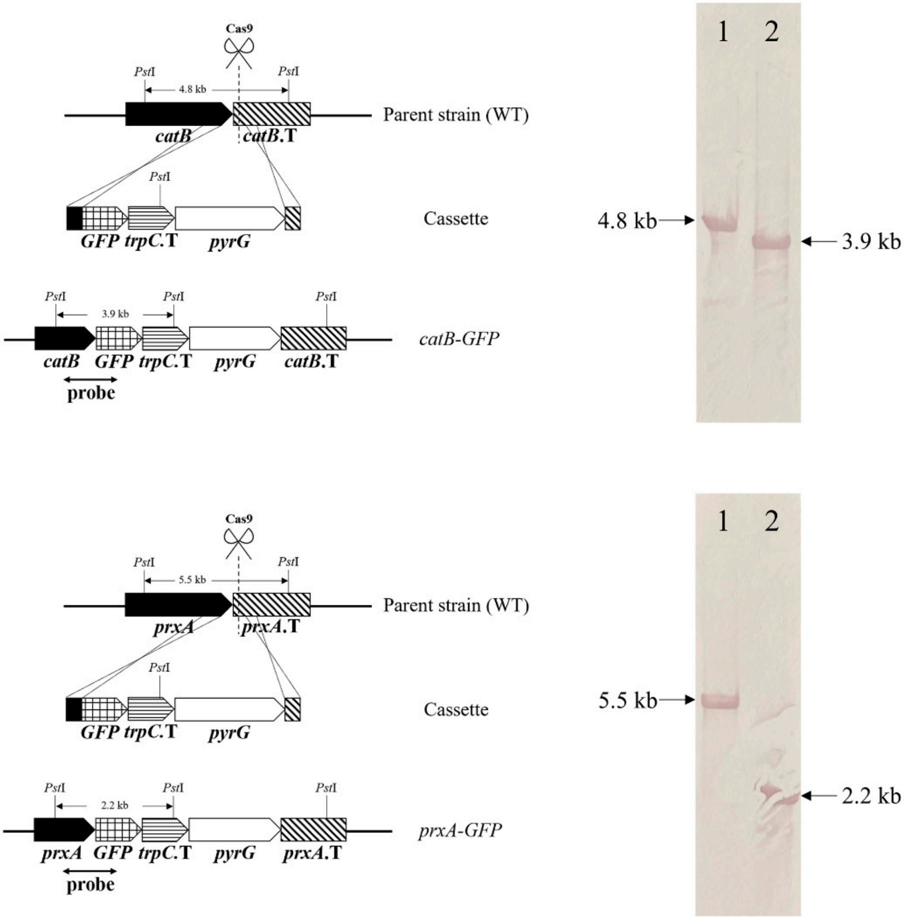

Figure S8. Identification by Southern blotting analyses of *A. nidulans* transformants carrying the *catB-GFP* or *prxA-GFP* genotype. Strategy of the cleavage site and probes was shown on the left, and the verification results were shown on the right. The binding results of *Pst*I digested fragments of the transformants genomes with probes were shown in lane 1 and lane 2, respectively. Primers for the probes were listed in Supplementary Table 7.

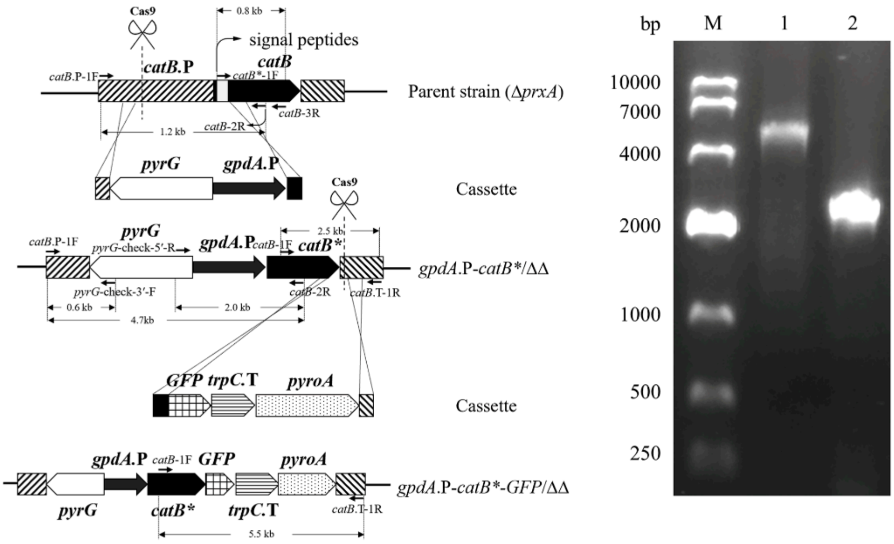

Figure S9. Construction of the strains overexpressing CatB* or CatB*-GFP in Δ*prxA*Δ*catB*. The construction and verification methods of *gpdA*.P-*catB**/ΔΔ were similar to that of *gpdA*.P-*catB**/WT (left). *gpdA*.P-*catB**-*GFP*/ΔΔ was constructed using *gpdA*.P-*catB**/ΔΔ as its parent strain. To verify the construction of *gpdA*.P-*catB**-*GFP*/ΔΔ, the indicated primer pair *catB*-1F/*catB*.T-1R were used to amplify the corresponding region in the isolated transformant (lane 1) and the parent (lane 2) strains. The corresponding primers were listed in Supplementary Table 6.

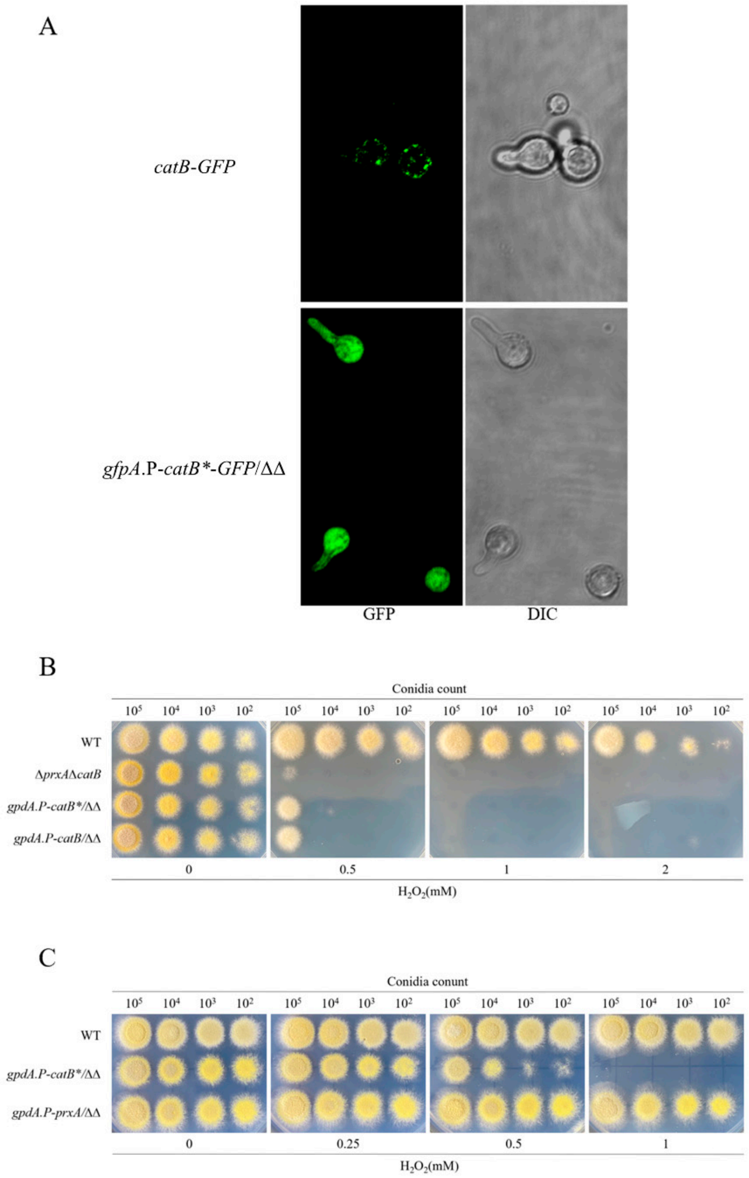

Figure S10. Cytosolic CatB cannot functionally substitute PrxA at the swelling and germinating conidia stage. A, *GFP* fused N-terminal signaling peptide absent *catB* (*catB*-GFP*) can be overexpressed in cytosol under the control of *gpdA* promoter in Δ*prxA*Δ*catB* (*gpdA.*P-*catB*-GFP*/ΔΔ). Top panel, *catB-GFP* expressed in WT under the control of the native *catB* promoter. Bottom panel, *catB**-*GFP* expressed in Δ*prxA*Δ*catB* (ΔΔ) under the control of *gpdA* promoter. After 5 h cultivation of the conidia of *catB-GFP* and *catB**-*GFP*, the fluorescence from the swelling and germinating conidia were analyzed via fluorescence microscopy. B, phenotypes of WT, Δ*prxA*Δ*catB*, *gpdA.*P-*catB**/ΔΔ and *gpdA.*P-*catB*/ΔΔ at the swelling and germinating conidia stage under H_2O_2 stress conditions. C, phenotypes of WT, *gpdA.*P-*catB**/ΔΔ and *gpdA.*P-*prxA*/ΔΔ at the swelling and germinating conidia stage under H_2O_2 stress conditions. Conidia from these strains were spotted on the plates containing the indicated concentrations of H_2O_2 to compare their H_2O_2 resistance.

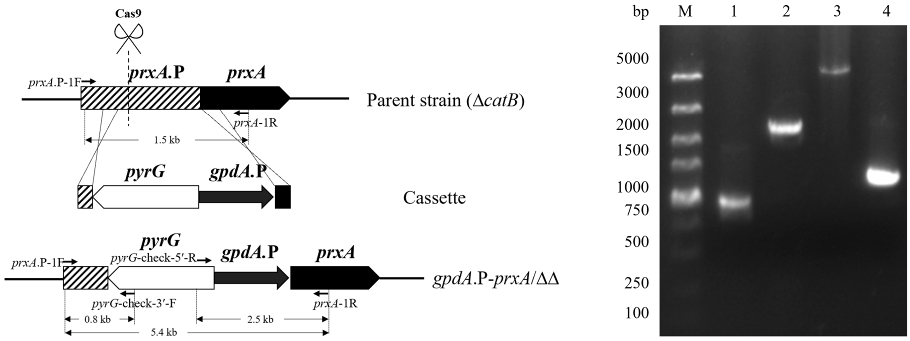

Figure S11. Construction of *prxA* promoter was replaced by *gpdA* promoter strains in *A. nidulans*. Promoter replacement strategies for homologous recombinant strains *gpdA*.P-*prxA*/ΔΔ was shown on the left. Validation of the corresponding recombination by PCR with the indicated primers was shown on the right. The *gpdA*.P-*prxA*/ΔΔ was verified with the primer pairs *prxA*.P-1F/*pyrG*-check-3′-F (lane 1) and *prxA*-1R/*pyrG*-check-5′-R (lane 2). The primer pair *prxA*.P-1F/*prxA*-1R was used to amplify the corresponding regions in the isolated transformant (lane 3) and parent (lane 4) strains. The corresponding primers were listed in Supplementary Table 6.
