# Supplementary material for: Cytosol Peroxiredoxin and Cell Surface Catalase Differentially Respond to H2O2 Stress in Aspergillus nidulans
Source: Antioxidants (Basel). 2023 Jun 23;12(7):1333. doi: 10.3390/antiox12071333 (PMC10376852; doi:10.3390/antiox12071333)
Supplement: Supplementary file 1 [file antioxidants-12-01333-s001.zip › Supplementary table_Final version.pdf]

Supplementary Table S1 Strains used in this study

Strains	Genotype	Source
WT	*biA1;argB2;pyrG89;wA3;pyroA4*	Our Lab
Δ*catB*	*biA1;argB2;pyrG89;wA3;pyroA4;ΔcatB::argB*	Our Lab
Δ*prxA*	*biA1;argB2;pyrG89;wA3;pyroA4;ΔprxA::argB*	Our Lab
Δ*catB*Δ*prxA*	*biA1;argB2;pyrG89;wA3;pyroA4;*<br>*ΔprxA::argB;ΔcatB::pyrG*	This work
Δ*prxA-prxA*^{com}	*biA1;argB2;pyrG89;wA3;pyroA4;*<br>*ΔprxA::argB; ΔpyrG:: prxA*^{com}*:pyrG*	This work
Δ*catB-catB*^{com}	*biA1;argB2;pyrG89;wA3;pyroA4;*<br>*ΔcatB::argB; ΔpyrG:: catB*^{com}*:pyrG*	This work
catB-GFP	*biA1; argB2;pyrG89;wA3;pyroA4;ΔcatB::catB:GFP:pyrG*	This work
catB-flag	*biA1; argB2;pyrG89;wA3;pyroA4;ΔcatB::catB:flag:pyrG*	This work
prxA-GFP	*biA1; argB2;pyrG89;wA3;pyroA4;ΔprxA::prxA:GFP:pyrG*	This work
catB.P-*uidA*	*biA1;argB2;pyrG89;wA3;pyroA4;*<br>*ΔpyrG::catB*.P*:uidA:pyrG*	This work
prxA.P-*uidA*	*biA1;argB2;pyrG89;wA3;pyroA4;*<br>*ΔpyrG::prxA*.P*:uidA:pyrG*	This work
gpdA.P-DAAO/WT	*biA1;argB2;pyrG89;wA3;pyroA4;*<br>*ΔpyrG::gpdA*.P*:*DAAO*:pyrG*	This work
gpdA.P-DAAO/Δ*prxA*	*biA1;argB2;pyrG89;wA3;pyroA4;*<br>*ΔpyrG::gpdA*.P*:*DAAO*:pyrG; ΔprxA::pyroA*	This work
gpdA.P-DAAO/Δ*catB*	*biA1; argB2;pyrG89;wA3;pyroA4;*<br>*ΔpyrG::gpdA*.P*:*DAAO*:pyrG; ΔcatB::pyroA*	This work
gpdA.P-DAAO/ΔΔ	*biA1; argB2;pyrG89;wA3;pyroA4;*<br>*ΔpyrG::gpdA*.P*:*DAAO*:pyrG; ΔcatB::pyroA; ΔprxA::argB*	This work
prxA.P-*catB*/ΔΔ	*biA1;argB2;pyrG89;wA3;pyroA4;*<br>*ΔprxA::argB;ΔcatB*.P*::pyrG:prxA*.P	This work
gpdA.P-*catB*/ΔΔ	*biA1;argB2;pyrG89;wA3;pyroA4;*<br>*ΔprxA::argB;ΔcatB*.P*::pyrG:gpdA*.P	This work
gpdA.P-*catB*/WT	*biA1; argB2;pyrG89;wA3;pyroA4;ΔcatB*.P*::pyrG:gpdA*.P	This work
gpdA.P-*catB**/WT	*biA1; argB2;pyrG89;wA3;pyroA4;*<br>*ΔcatB*.P*::pyrG:gpdA*.P*:catB**	This work
gpdA.P-*catB**/ΔΔ	*biA1; argB2;pyrG89;wA3;pyroA4;*<br>*ΔprxA::argB;ΔcatB*.P*::pyrG:gpdA*.P*:catB**	This work
gpdA.P-*catB**-<br>*GFP*/ΔΔ	*biA1; argB2;pyrG89;wA3;pyroA4;ΔprxA::argB;*<br>*ΔcatB*.P*::pyrG:gpdA*.P*:catB*;ΔcatB::catB:GFP:pyroA*	This work
gpdA.P-*prxA*/ΔΔ	*biA1;argB2;pyrG89;wA3;pyroA4;*<br>*ΔcatB::argB;ΔprxA*.P*::pyrG:gpdA*.P	This work

*catB**: *catB* lack of N-signal peptides.

Supplementary Table S2 Plasmids used in this study

Plasmid	Source
pUC19-*pyrG*	Our Lab
pUC-*GFP*	Our Lab
pUC19-*GFP*-*trpC*.T-*pyrG*	This work
pUC19-*GFP*-*trpC*.T-*pyroA*	This work
pUC19-*pyrG*-*catB*.P-*uidA*-*trpC*.T	This work
pUC19-*pyrG*-*prxA*.P-*uidA*-*trpC*.T	This work
pUC19-*pyrG*-*gpdA*.P-*uidA*-*trpC*.T	This work
pUC19-*prxA*^{com}-*pyrG*	This work
pUC19-*catB*^{com}-*pyrG*	This work
pUC19-*pyrG*-*gpdA*.P-DAAO-*trpC*.T	This work

Supplementary Table S3 Primers used for plasmids construction

Primers	Template	Nucleotide sequence (5' to 3')
Inverse-1F	pUC19-*pyrG*	GGCGTAATCATGGTCATAGC
Inverse-1R		CCTGCAGGTCGACTCTAGAG
GFP-F	pUC-*GFP*	GCTATGACCATGATTACGCCGGAGCTGGTGCA GGCGCTGG
GFP-R		CTACACGTGATGATGATGATGATGCATTTTGTA TAGTTCATCCATGCCATG
trpC.T-1F	A6 gDNA	ATGCATCATCATCATCATCACGTGTAGTAGTGA TTTAATAGCTCC
trpC.T-1R		CTCTAGAGTCGACCTGCAGGAAAGAAGGATTA CCTCTAAAC
Inverse-2F	pUC19-*GFP*-*trpC*.T-*pyrG*	TCTAGAGGATCCCCGGGTAC
Inverse-2R		CTAGAGTCGACCTGCAGGCATG
pyroA-F	A6 gDNA	CCTGCAGGTCGACTCTAGCGAGCTCGAATTCG GATCCAG
pyroA-R		ACCCGGGGATCCTCTAGACTGCAGAAGTGCGC GAAAGC
catB.P-F	A6 gDNA	CTCTAGAGTCGACCTGCAGGTGTAGAATATCAT GTAGAG
catB.P-R		GGTTTCTACAGGACGTAACATGGTGAGGTAAA GTCAAGATAAAG
trpC.T-2F	A6 gDNA	AGCAGGGAGGCAAACAATGATAGTGATTTAAT AGCTCCATG
trpC.T-2R		GCTATGACCATGATTACGCCAAAGAAGGATTA CCTCTAAAC
uidA-F	*E. coli* BL21	ATGTTACGTCCTGTAGAAAC
uidA-R		TCATTGTTTGCCTCCCTGCTG
prxA.P-F	A6 gDNA	CTCTAGAGTCGACCTGCAGGGGTTACGACTTC TGGAAAAC
prxA.P-R		GGTTTCTACAGGACGTAACATTGTGTAAGATA GTGGTTGTTGG
gpdA.P-F	A6 gDNA	CTCTAGAGTCGACCTGCAGGGCTCCTTATTGA AGTCGGAG
gpdA.P-R		GGTTTCTACAGGACGTAACATTGTGATGTCTGC TCAAGCGG
prxA^{com}-F	A6 gDNA	TTGATTTCGAGCTTATGGTTCC
prxA^{com}-R		TCCTCGATCATCTATTCCCGTC
catB^{com}-F	A6 gDNA	CTTGCTCAGGAGGTTCCGGTTG
catB^{com}-R		CGGTCATGACTCGTTGCTTG
DAAO-F	*Rhodotorula gracilis*	ATGCACTCCCAGAAGCGCGTC
DAAO-R		TTAGGACTCGCGGGCGGCAC

Supplementary Table S4 Primers used for sgRNA synthesis

Primers	Gene location	Nucleotide sequence (5' to 3')
catB-sgF	*catB*	TAATACGACTCACTATAGAGGAGGACCAGTTGCGCTT
catB-sgR		TTCTAGCTCTAAAACAAGCGCAACTGGTCCTCCTC
catB.T-sgF	*catB*.T	TAATACGACTCACTATAGACGCCCGCTAACGAGGACT
catB.T-sgR		TTCTAGCTCTAAAACAGTCCTCGTTAGCGGGCGTC
prxA.T-sgF	*prxA*.T	TAATACGACTCACTATAGTATAAGAGGGCACCCTTGT
prxA.T-sgR		TTCTAGCTCTAAAACACAAGGGTGCCCTCTTATAC
Insert-sgF	*AN*9329-*AN*9328	TAATACGACTCACTATAGACACGTCCAGTCCTTACTA
Insert-sgR		TTCTAGCTCTAAAACTAGTAAGGACTGGACGTGTC
catB.P-sgF	*catB*.P	TAATACGACTCACTATAGGCAGGGCTCATCAATGTGA
catB.P-sgR		TTCTAGCTCTAAAACTCACATTGATGAGCCCTGCC

Supplementary Table S5 Primers used for strains construction

Primer	Template	Nucleotide sequence (5' to 3')
**Gene Disruption**		
catB-F	pUC19-*pyrG*	CAGCGCCCTTTACCCGACCTCAAGCCCGACCTCCTCTGCATTCCTATCCCTCTTTCCTAAAAACCACCAACTTTTATCTTTATCTTGACTTTACCTCACCGAATTCGATACCTGTCGAAAG
catB-R	pUC19-*pyrG*	CAGTGACGATTATAGGACAGTTTGTCAAATTTGAAAATCAATAAATATAAATATATTATCTATACAGATAAGCTCTCGGCTACTAGTCTCATTCTACCATTCAGTGCTTGTCTACCAG
**Tagged Expression**		
catB-*GFP*-F	pUC19-*GFP*-*trpC*.T-*pyrG*	GTACGTTGCCGATGCCGTGGACGAGAGCTTTGCCAACAACCTCGAGGAGGGTCTGACCGTGTTCAAGTTCTTGGATCGCTTTGCCCTGGACTCGGATGAAGGAGCTGGTGCAGGCGCTGG
catB-*GFP*-R	pUC19-*GFP*-*trpC*.T-*pyrG*	ACACATTGCTGTCACTTAAGTTTTGTAAGAATGTGACTGGCATTCCTACCCAAGCACGCGGCGGCAACAAGGGAACCCCGACACCGCACGAGGTATGTGGGATCAGTGCTTGTCTACCAG
prxA-*GFP*-F	pUC19-*GFP*-*trpC*.T-*pyrG*	TGCCATCGTCCTCGACCACGGCAAGGTCACCTACGCTGCTCTGGAGCCCGCCAAGAACCACCTTGAGTTCTCCAGCGCCGAGACTGTCATCAAGCACCTGGGAGCTGGTGCAGGCGCTGG
prxA-*GFP*-R	pUC19-*GFP*-*trpC*.T-*pyrG*	GTGAAACTTAGAGCTCCATTGGGAAATTGGATTGGCAAGACTGGGCTTGATAGGGCCTGGGTTCCGTGTTGAATGGAACTTTTTAGCGCGTCCGCCGCTGGATCAGTGCTTGTCTACCAG
catB-*flag*-F	pUC19-*GFP*-*trpC*.T-*pyrG*	GCTTTGCCAACAACCTCGAGGAGGGTCTGACCGTGTTCAAGTTCTTGGATCGCTTTGCCCTGGACTCGGATGAAGATTACAAGGACGACGATGACAAGATGCATCATCATCATCATCACG
catB-*flag*-R	pUC19-*GFP*-*trpC*.T-*pyrG*	ACACATTGCTGTCACTTAAGTTTTGTAAGAATGTGACTGGCATTCCTACCCAAGCACGCGGCGGCAACAAGGGAACCCCGACACCGCACGAGGTATGTGGGATCAGTGCTTGTCTACCAG
*catB**-*GFP*-R	pUC19-*GFP*-*trpC*.T-*pyroA*	ACACATTGCTGTCACTTAAGTTTTGTAAGAATGTGACTGGCATTCCTACCCAAGCACGCGGCGGCAACAAGGGAACCCCGACACCGCACGAGGTATGTGGCTGCAGAAGTGCGCGAAAGC
**GUS Expression**		
catB.P-*uidA*-F	pUC19-*pyrG*-*catB*.P-*uidA*-*trpC*.T	ACTCCTGTGAAATGAGTATGATGAAGTTGATGGGCAGTGTTTGAATGTGACGGTGACTTATGAATCCCAACCACCATAATAATAAGACTGCGCAATCTCCTCAGTGCTTGTCTACCAG
catB.P-*uidA*-R	pUC19-*pyrG*-*catB*.P-*uidA*-*trpC*.T	CCTGGTAATGGTCTTCAAAACCTAGCGACTACAGAACGCGAAACTCCTGAAGTATTTGGGTATTTGGGTATGAGAGAGTGCGCCTTGGGGGTGCATGCGCAAAGAAGGATTACCTCTAAAC
prxA.P-*uidA*-F	pUC19-*pyrG*-*prxA*.P-*uidA*-*trpC*.T	ACTCCTGTGAAATGAGTATGATGAAGTTGATGGGCAGTGTTTGAATGTGACGGTGACTTATGAATCCCAACCACCATAATAATAAGACTGCGCAATCTCCTCAGTGCTTGTCTACCAG
prxA.P-*uidA*-R	pUC19-*pyrG*-*prxA*.P-*uidA*-*trpC*.T	CCTGGTAATGGTCTTCAAAACCTAGCGACTACAGAACGCGAAACTCCTGAAGTATTTGGGTATTTGGGTATGAGAGAGTGCGCCTTGGGGGTGCATGCGCAAAGAAGGATTACCTCTAAAC

Primer	Template	Nucleotide sequence (5' to 3')
		**CatB Overexpression**
prxA.P-*catB*-F	pUC19-*pyrG*-*prxA*.P-*uidA*-*trpC*.T	CAGGTCCCCAACCACTCATCAGACCATAATTACTGATAGCCAAGCAGATCGCCTGCTCGGCACCACCTGCTTAACCCCGAATAAGACATCGCTCCGAGCGTCAGTGCTTGTCTACCAG
prxA.P-*catB*-R		TAGCATCGGGGTTGGTATCGCGACGGCCGAGCTCGCCTGTCATATACGGACAGACGGCATTGGCGACGCCAACAAGGCCGACCAGGCCGAGAGCTCGCATTGTGTAAGATAGTGGTTGTTG
gpdA.P-*catB*-F	pUC19-*pyrG*-*gpdA*.P-*uidA*-*trpC*.T	CAGGTCCCCAACCACTCATCAGACCATAATTACTGATAGCCAAGCAGATCGCCTGCTCGGCACCACCTGCTTAACCCCGAATAAGACATCGCTCCGAGCGTCAGTGCTTGTCTACCAG
gpdA.P-*catB*-R		TAGCATCGGGGTTGGTATCGCGACGGCCGAGCTCGCCTGTCATATACGGACAGACGGCATTGGCGACGCCAACAAGGCCGACCAGGCCGAGAGCTCGCATTGTGATGTCTGCTCAAGCGG
gpdA.P-*catB**-F	pUC19-*pyrG*-*gpdA*.P-*uidA*-*trpC*.T	CAGGTCCCCAACCACTCATCAGACCATAATTACTGATAGCCAAGCAGATCGCCTGCTCGGCACCACCTGCTTAACCCCGAATAAGACATCGCTCCGAGCGTCAGTGCTTGTCTACCAG
gpdA.P-*catB**-R		TGGGCCGCCGACGTCAGTCGTCAGGTACGAGTCCGTGTCGTCAAGGTAGTACTCGGACAGAAATTCCTCAGTGGCCTCGGTAGCATCGGGGTTGGTATCCATTGTGATGTCTGCTCAAGCGG
		**PrxA Overexpression**
gpdA.P-*prxA*-F	pUC19-*pyrG*-*gpdA*.P-*uidA*-*trpC*.T	CTCGACGGTCTTTCTTGATAATACAGGCGGCGAATCATCGAAAGGATTCGTTCGGCATCCTTCTGCTTCGGATCACCTCACTACGGAGTATGGATGGCGCTCAGTGCTTGTCTACCAG
gpdA.P-*prxA*-R		TCAATTGTACTAATAAGAATTTAGAGCAACATACGAGAAGACAACGTCAGCGGGGAAGCTGTCACCGGCCTTAAGTCCAGACATTGTGATGTCTGCTCAAGCGG

Supplementary Table S6 Primers used for strains verification

Primers	Gene location	Nucleotide sequence (5’ to 3’)
pyrG-check-5′-R	*pyrG*	TAGGAGAGACCCGTGTTCCG
pyrG-check-3′-F	*pyrG*	CTCACTGACGGCTCTAGTAG
catB.P-1F	*catB*.P	CGTCGAGACTGGTTGTATATCC
catB-1F	*catB*	CCACCAAGCTCGTCAAGTTCC
catB-1R	*catB*	AGAGGGTTGAGCTGGAGTTGG
catB.T-1R	*catB*.T	CGGTCATGACTCGTTGCTTG
prxA.P-1F	*prxA*.P	CTATTAAGGATACGTCTGGCG
prxA-1F	*prxA*	CCCCGCTGACGTTGTCTTC
prxA-1R	*prxA*	GGTCGAGGACGATGGCATAAC
prxA.T-1R	*prxA*.T	GCTCTAGCCCTCAATCTCCTC
AN9329-1F	*AN9329*	TCGACTGGCCTAAAGTCCCTG
uidA-1F	*uidA*	ACAGCGAAGAGGCAGTCAACG
AN9328-1R	*AN9328*	GCTGGCTAGTTCCGTCTTCTC
catB-2R	*catB*	GGCCTTGAGACTCTGCTGG
*catB**-1F	*catB*	TCCGTATATGACAGGCGAGC
catB-3R	*catB*	GGAACTTGACGAGCTTGGTGG
gpdA.P-1F	*gpdA*.P	CGAGATAGTACCTTCTCCG
DAAO-1R	DAAO	CTCGACCCACTTCTTGAAG

Supplementary Table S7 Primers used for probe synthesis

Primers	Probe	Nucleotide sequence (5' to 3')
uidA-probe-F	*uidA*	CCTGTGGGCATTCAGTCTGG
uidA-probe-R	*uidA*	CTTCACTGCCACTGACCGGATG
catB-probe-F	*catB*	GCCGTACTTGCTTCTGTTGAC
GFP-probe-R	*GFP*	TCTTCGATGTTGTGGCGGGTC
prxA-probe-F	*prxA*	ACTGAGGAGAAGGGCGAGATC

Supplementary Table S8 Primers used for Q-RT-PCR

Primers	Gene	Nucleotide sequence (5’ to 3’)
RT-*actA*-F	*actA*	GAAGTCCTACGAACTGCCTGATG
RT-*actA*-R		GACCAAGAACGCTGGGCTGG
RT-*catB*-F	*catB*	CAACCCCGATGCTACCGAG
RT-*catB*-R		GGCCTTGAGACTCTGCTGG
